# Brain Metastases in Oncogene-Addicted Non-Small Cell Lung Cancer Patients: Incidence and Treatment

**DOI:** 10.3389/fonc.2018.00088

**Published:** 2018-04-11

**Authors:** J. Remon, Benjamin Besse

**Affiliations:** ^1^Medical Oncology Department, Hospital Universitari Vall d’Hebron, Barcelona, Spain; ^2^Cancer Medicine Department, Institut Gustave Roussy, Villejuif, France; ^3^University Paris-Sud, Orsay, France

**Keywords:** brain, metastases, non-small cell lung cancer, EGFR, ALK

## Abstract

Brain metastases (BM) are common in non-small cell lung cancer patients including in molecularly selected populations, such as *EGFR*-mutant and *ALK*-rearranged tumors. They are associated with a reduced quality of life, and are commonly the first site of progression for patients receiving tyrosine kinase inhibitors (TKIs). In this review, we summarize incidence of BM and intracranial efficacy with TKI agents according to oncogene driver mutations, focusing on important clinical issues, notably optimal first-line treatment in oncogene-addicted lung tumors with upfront BM (local therapies followed by TKI vs. TKI monotherapy). We also discuss the potential role of newly emerging late-generation TKIs as new standard treatment in oncogene-addicted lung cancer tumors compared with sequential strategies.

## Introduction

Lung cancer is the leading cause of cancer-related deaths worldwide (1). Because of the lack of screening programs in most countries, more than half of non-small cell lung cancer (NSCLC) patients are diagnosed at an advanced stage^1^. The brain is a common metastatic site in this population, with 30% of patients developing brain metastases (BM) during the course of their disease, with the brain being the only site of metastatic disease in 51% of these cases. Median delay between diagnosis of the primary tumor and development of BM is 11 months. Up to half of cases, patients present with synchronous diagnosis of BM at the time of diagnosis of the primary lung tumor (2). Ironically, the lifetime incidence of BM is increasing due to prolonged survival seen in NSCLC patients thanks to new systemic therapies and improved neuro-imaging techniques (3). Unfortunately, prognosis associated with BM remains poor with reports of median overall survival (OS) between 3 and 14.8 months (4), and compared to other metastatic sites, BM are responsible for a major decrease in quality of life (5).

The discovery of targetable genomic alterations in approximately 30% of advanced NSCLC tumors, mainly adenocarcinomas, has altered the therapeutic landscape and outcome of many of these subgroups of NSCLC patients (6, 7). In the recent era of personalized treatment targeting these alterations, prognosis of NSCLC patients with BM has improved significantly achieving a median OS of nearly 4 years (8). The question whether BM harbor distinct genetic alterations beyond those observed in primary tumors has not been definitively addressed. Recent data with whole-exome sequencing in 86 patient-matched BM (including 38 NSCLC patients) reported 53% of cases with potentially clinically informative alterations in BM that were not detected in the matched primary-tumor sample (9). However, these findings have a number of technical limitations and are yet to be supported by clinical evidence. On the other hand, response rates (RR) to targeted therapies in molecularly defined NSCLC patients are typically similar in central nervous system (CNS) and extra-CNS disease, arguing for fewer molecular discordances between the primary tumor and CNS metastases, at least for actionable mutations. This is an important issue to resolve for determining the best treatment strategies for managing BM.

One important consideration, when interpreting CNS efficacy with tyrosine kinase inhibitors (TKIs) in molecularly selected NSCLC patients, is the inherent limitation of the standard RECIST criteria for the measurement of baseline CNS disease and response (10). This assessment does not account for potential pseudo-progression correlating with radionecrosis and non-viable tumors in patients who have received brain radiotherapy (11). New imaging tests might offer better characterization of CNS progression vs. pseudo-progression (12). While the systemic efficacy of TKI in oncogene-addicted NSCLC has been well established, their intracranial efficacy is today less well validated for a number of reasons. Brain imaging during follow-up is often optional in clinical trials, MRI is not commonly used compared to the less sensitive CT scan, and patients with BM are often excluded from NSCLC trials, and when they are accepted, BM is not a stratification criteria. The CNS is shielded by the blood–brain barrier (BBB) and is considered a “pharmacological sanctuary.” The key molecular properties that influence the BBB are the P-glycoprotein (P-gp) or breast cancer resistance protein substrate nature of the TKIs, their molecular weight, polar surface area and lipophilicity index (LogP) (13). These factors may explain why only 2% of small-molecule drugs are able to effectively cross the BBB (14), likely explaining why the CNS is a frequent site of failure after clinical benefit with some TKIs.

In this review, we summarize the incidence of BM in oncogene-addicted NSCLC patients and CNS efficacy for personalized treatment in these different sub-populations. We also evaluate new challenges such as the value of upfront personalized treatment vs. radiotherapy in oncogene-addicted NSCLC patients with BM at baseline, and administration of more potent drugs upfront vs. sequential treatment.

## EGFR-Mutant NSCLC Patients

Within the lung cancer population, activated epidermal growth factor receptor (*EGFR)* mutations occur in 10% of Caucasians and 50% of Asians (15). There are several classes of activating somatic *EGFR* mutations, with in-frame deletions in exon 19 (ELREA, *Del1*9) and single-point mutations in exon 21 (*L858R*) being the most common. These mutations predict sensitivity to first- and second-generation EGFR TKIs, such as erlotinib, gefitinib, or afatinib. RRs and progression-free survival (PFS) with EGFR TKIs have proven superior to standard first-line platinum doublet chemotherapy, making them the current upfront standard of care (16). Recently, osimertinib a third-generation EGFR TKI, showed a significant improvement in PFS compared with standard of care (erlotinib or gefitinib) as first-line treatment, making it a new treatment option in the first-line setting (17).

### Incidence of BM in EGFR-Mutant NSCLC

The baseline incidence of BM in *EGFR*-mutant NSCLC is similar to that of other oncogenic driver mutations, ranging from 23 to 32% (18–20). The cumulative incidence increases over time (19, 21), with a 2-year actuarial risk of CNS progression of approximately 15–20% when patients received standard of care EGFR TKIs (21, 22). BM development on EGFR TKI treatment is significantly more common among patients with baseline BM (2-year cumulative incidence of 47% among patients with pre-existing BM compared to 11% among those with no prior BM; *p* = 0.003) and correlates with a worse outcome (21, 23, 24). Literature reporting the risk of cumulative incidence of brain progression according to *EGFR* mutation subtype is contradictory, some studies reporting higher cumulative risk among *Del19-*mutant tumors (21), and others among *L858R*-mutant tumors (22, 24).

Although, it has been suggested that *EGFR* mutations appear early during multistep carcinogenesis and may even be associated with an increased propensity for metastatic cell to spread into the brain (25), the lifetime risk is confounded by this molecular subgroup’s longer survival. However, some reports suggest that the incidence of BM is higher in *EGFR*-mutant patients compared to *EGFR*-wild type (31.4 vs. 19.7%, odds ratio 1.86, 95% CI: 1.39–2.49; *p* < 0.001) (18), but it could be explained by inability of first-generation EGFR TKIs to cross BBB, reported in up to 60% of patients (26, 27). The high incidence and significant rate of CNS failure highlights the need for additional strategies to prevent CNS progression.

### Treatment With EGFR TKIs

First- and second-generation EGFR TKI brain penetration potential, measured by the unbound brain-to-plasma ratio, termed K_*p*,uu_, is very low (28), indicating that penetration into the brain is diffusion-limited or low passive BBB permeability (13). However, the importance of the BBB for intracranial tumors is debated. Retrospective observational and phase II studies have reported activity with erlotinib and gefitinib in *EGFR*-mutant patients with BM (29–34). Two studies with erlotinib showed intracranial RRs of 58 and 82% and intracranial PFS of 10.1 and 11.7 months (29, 30). Gefitinib achieved an intracranial RR of 88% and intracranial PFS of 14.5 months, with a time to salvage brain radiation from diagnosis of 17.9 months (32) (Table 1).

**Table 1:** Efficacy of EGFR tyrosine kinase inhibitors (TKIs) in *EGFR*-mutant non-small cell lung cancer (NSCLC) patients and brain metastases (BM).

Drug	Trial	*N*	icRR (%)	icDOR (months)	icPFS (months)
Erlotinib	Retrospective (29)	17	82	NA	11.7
	Ph II (30)	8	58.4	NA	10.1
Gefitinib	Ph II (32)	41	88	NA	14.5
	Retrospective (34)	14	43	7.7	9.1
Afatinib	Pooled analysis (37)	81	21^a^	NA	8.2^a^
Icotinib^b^	Ph III (38)	85	65	NA	10.0
AZD3759	Ph I (28)	18	83	NA	NA
Osimertinib	AURA + AURA2 (49, 50)	128	54^c^	NR	1 year: 56%
	AURA3 (51)	116	70^d^	8.9^d^	11.7
	FLAURA (17)	128	66	NA	NR

*icDOR, intracranial duration of response; icRR, intracranial response rate; icPFS, intracranial progression-free survival; NA, not available; NR, not reached*.

*^a^Systemic RR and progression-free survival (PFS)*.

*^b^Patients should have at least 3 metastatic brain lesions*.

*^c^In 50 evaluable patients*.

*^d^In 30 evaluable patients with osimertinib*.

*In vivo* studies in NSCLC mice showed that afatinib penetrated the BBB and cerebrospinal fluid (CSF) levels correlated with plasma levels (35). In a compassionate-use program including 31 patients, afatinib demonstrated a 35% CNS response in molecularly non-selected patients who had previously failed TKI therapy, with a median time to treatment failure of 3.6 months (36). In a combined dataset *post hoc* analysis in 81 *EGFR*-mutant NSCLC patients with BM (30% had received brain radiotherapy) in the first-line LUX-Lung 3 and LUX-Lung 6 phase III clinical trials, afatinib significantly improved PFS (8.2 vs. 5.4 months, hazard ratio (HR) 0.50, *p* = 0.0297) and RR (21 vs. 5%, *p* = 0.0027), although without a significant difference in OS (22.4 vs. 25.0 months; HR 1.14, *p* = 0.64) compared with platinum-based chemotherapy (37). The magnitude of the PFS benefit was suggested being increased, for patients who had received prior whole brabin radiotherapy (WBRT, *n* = 24; 13.8 vs. 4.7 months; HR 0.37, *p* = 0.07). Evaluation of intracranial response was not assessed as a separate endpoint in these trials (37), however, these results suggest that asymptomatic BM are not a limitation for upfront treatment with an EGFR TKI (Table 1).

Icotinib, another EGFR TKI only available in Asia, gave an intracranial RR of 65% and median PFS of 10 months in treatment-naïve *EGFR*-mutant patients with at least three BM (38). AZD3759 is a novel reversible EGFR TKI, only active against sensitizing *EGFR* mutations, which was designed to effectively cross the BBB and achieves high drug-free exposure in the brain. In a phase I trial, it achieved an intracranial RR of 83% among 18 EGFR TKI treatment-naïve patients with evaluable BM (28) (Table 1).

The substitution of threonine to methionine at amino acid position 790 (*T790M*) in exon 20 of the *EGFR* gene reduces first-generation EGFR TKI binding by enhancing the ATP binding affinity of the kinase domain of the *EGFR*-mutant receptor (39). It accounts for acquired resistance in approximately 50–60% of patients (40, 41). Knowledge of acquired resistance mechanisms to EGFR TKIs was one of the triggers behind the development of third-generation EGFR-TKIs, such as osimertinib, which are active against *exon19* and *21* mutations as well as the *T790M* mutations. Osimertinib was the first such agent to receive FDA and EMA approval (in November 2015 and February 2016, respectively) for metastatic *EGFR*-mutant and acquired *EGFR T790M*-mutant NSCLC patients progressing on or after EGFR TKI therapy^2^^,^^3^.

The rate of acquired *T790M* mutations is discordant between intracranial and extracranial metastases. In a study of 78 *EGFR*-mutant patients who had undergone re-biopsy after TKI failure, only 17% of CNS lesions were *T790M* mutated compared to 41% of systemic lesions (42), suggesting that the selection pressure is lower intracranially owing to the lower EGFR TKI concentrations in CSF compared to serum concentrations (42, 43). Preclinical data demonstrated greater penetration and brain exposure with osimertinib than with gefitinib, rociletinib, or afatinib (44).

Central nervous system activity of osimertinib was reported in pretreated *T790M*-positive NSCLC patients in the AURA study phase II extension component (45), the phase II AURA2 trial (46), and was recently confirmed in the phase III AURA3 trial (47) and the first-line FLAURA trial (17). In the pooled analysis of the two phase II trials (*N* = 411), osimertinib demonstrated an overall RR of 66% and median PFS of 11 months (48). In the pre-specified subgroup analysis of CNS response in this pooled analysis among 128 patients with CNS metastases at baseline, 50 were evaluable for CNS response. CNS response and DCR were 54 and 92%, respectively, and CNS response was observed regardless of prior radiotherapy. Median CNS duration of response (DOR) was not reached and at 9 months 75% of patients were estimated to remain in response. Median CNS PFS was not reached (49), with 1-year PFS of 56% (50). In the AURA3 trial, osimertinib demonstrated significantly greater efficacy in RR (71 vs. 31%) and PFS (10.1 vs. 4.4, HR 0.30, 95% CI: 0.23–0.41, *p* < 0.001) than platinum-pemetrexed chemotherapy, in 419 *T790M*-positive NSCLC patients who had progressed on first-line EGFR TKIs (47). Among 116 patients from the AURA3 trial with BM (measurable or not), PFS was longer with osimertinib compared to chemotherapy (11.7 vs. 5.6 months, HR 0.32; 95% CI: 0.15–0.69) and cumulative incidence of CNS progression at 6 months was lower with osimertinib compared to chemotherapy (11.5 vs. 28.2%) (51). Among 46 patients with evaluable BM, the intracranial RR was 70% with osimertinib compared with 31% with chemotherapy, with a median DOR of 8.9 vs. 5.7 months, respectively (51) (Table 1). It has been proposed that BM may not develop secondary resistance mutations to EGFR TKIs that develop during extracranial progression, due to reduced drug penetration of the BBB (52). However, CNS efficacy with osimertinib reported in AURA3 trial, appears to contradict this theory. In the CNS full analysis set (*N* = 128) from FLAURA trial, osimertinib reported improved CNS RR (66 vs. 43%) and longer CNS PFS (NR vs. 13.9 months, HR 0.48, 95% CI: 0.26–0.86, *p* = 0.04) and reduced the risk of CNS progression compared with the standard of care. Among evaluable CNS evaluable patients (*N* = 41), osimertinib improved the CNS RR (91 vs. 68%) with similar DOR compared with the standard of care (15.4 vs. 18.7 months) (53).

In light of the reduced CSF concentrations of EGFR TKIs, various studies have examined administration of high doses in an attempt to achieve therapeutic levels (54–57). “Pulsatile” erlotinib at 1,500 mg given weekly resulted in an intracranial RR of 67% with a median time to CNS progression of 2.7 months in nine patients (55). In a phase I trial, pulse and daily low-dose erlotinib prevented progression of untreated or new CNS metastases, without improving extracranial outcome compared with standard-dose (58). However, the limited number of patients, the short follow-up period and the fact that half of the patients with baseline BM had already been treated are confounding factors that prevent any conclusions being reached regarding efficacy in the CNS with this strategy.

### Combined EGFR TKIs and Antiangiogenics

Activity and an acceptable safety profile of bevacizumab, an anti-VEGF monoclonal antibody, have been reported in NSCLC patients with asymptomatic and untreated BM (59). Moreover, in a preclinical model of lung adenocarcinoma, bevacizumab prevented BM formation (60). In a phase II trial in 154 Asian *EGFR*-mutant NSCLC patients, the addition of bevacizumab to erlotinib as first-line treatment significantly improved PFS compared to erlotinib alone (16.0 vs. 9.7 months, HR 0.54; 95% CI: 0.36–0.79, *p* = 0.0015) (61), leading to EMA approval of the combination in this population in April 2016. Two ongoing phase III trials evaluating erlotinib combined with ramucirumab (NCT02411448) or bevacizumab (BEVERLY study, NCT02633189) compared with erlotinib, will hopefully further validate this strategy.

In the single arm phase II BELIEF trial in 109 Caucasian *EGFR*-mutant NSCLC patients, combined erlotinib and bevacizumab gave median PFS and OS of 13.2 and 28.2 months, respectively. However, the primary endpoint was only met in baseline *T790M*-positive tumors with a median PFS of 16 months, whereas in *T790M*-negative tumors, median PFS was 10.5 months (62). In the subgroup of patients with pretreated BM (*N* = 21), median PFS was 8.8 months. The efficacy of this combination in the BM population does not appear to be superior to standard EGFR TKI therapy (37), however only 21 patients with BM were included in the BELIEF trial. Results from the ongoing randomized phase II BRILLANT trial (NCT0265536), testing bevacizumab plus erlotinib vs. erlotinib in BM *EGFR*-mutant patients, should reveal the efficacy of this combination in this population. Also the combination of osimertinib and bevacizumab in *EGFR*-mutant NSCLC patients and BM is currently assessed in a phase II trial (NCT02971501).

## ALK-Rearranged NSCLC Patients

**Figure 1:**
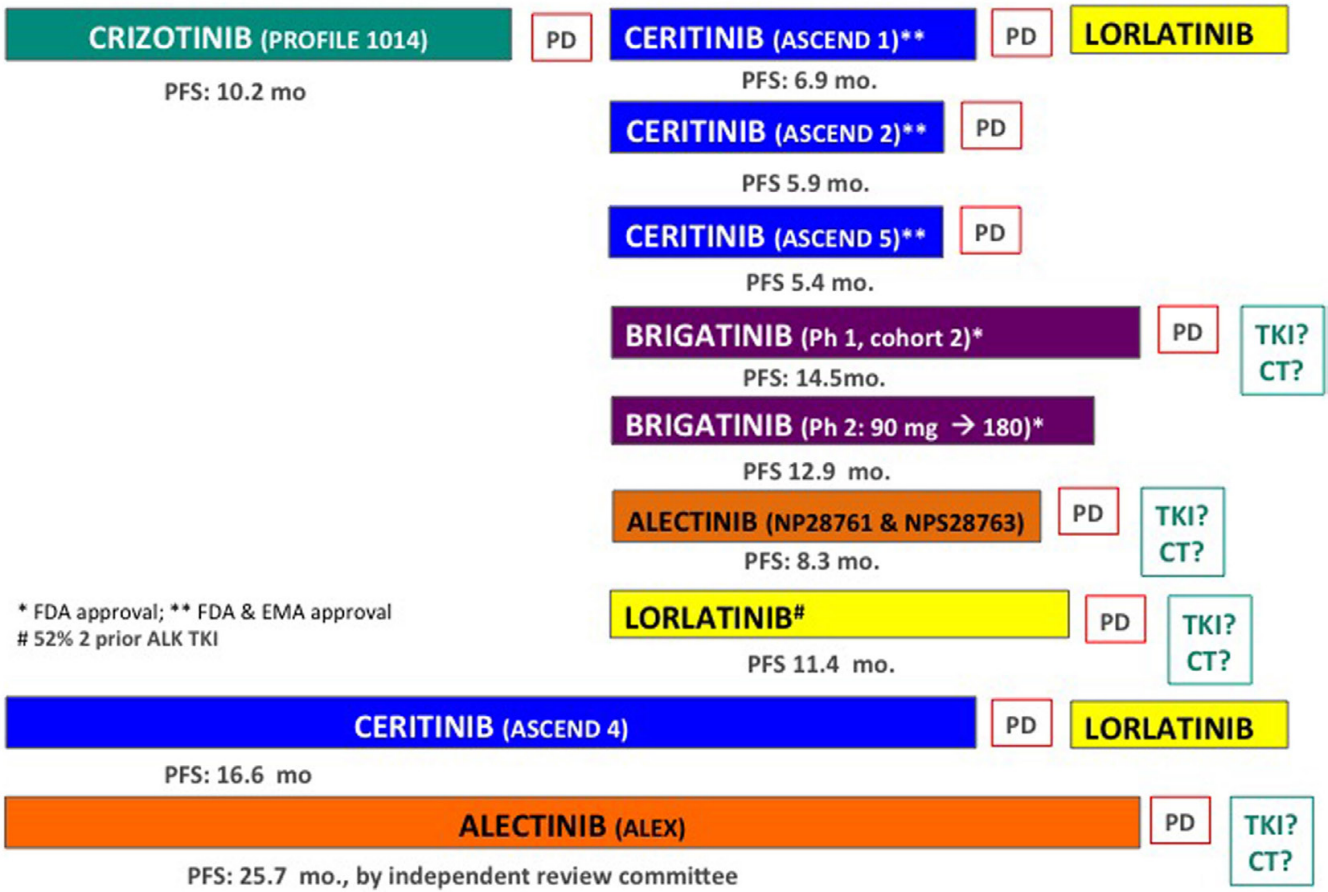
Systematic treatment stratergies in *ALK*-positive non-small cell lung cancer (NSCLC) patients. TKI, other ALK tyrosine kinase inhibitor; CT, chemotherapy.

Anaplastic lymphoma kinase (*ALK)* rearrangements result from inversions or translocations on chromosome 2 and are present in ~5% of NSCLC tumors, with no apparent differences in incidence according to race. Crizotinib was the first treatment to be approved in this population achieving a median PFS of 10.9 vs. 7.0 months with platinum-pemetrexed chemotherapy in the front-line setting in the phase III PROFILE 1014 study (63). In the subsequent phase III ASCEND-4 trial in *ALK*-positive (by central immunohistochemistry) NSCLC patients, upfront ceritinib, a second-generation ALK TKI, gave a median PFS of 16.6 vs. 8.8 months with platinum-pemetrexed chemotherapy (64). Based on these results, the FDA approved ceritinib as first-line treatment in *ALK*-positive NSCLC patients in May 2017. More recently, the phase III ALEX trial demonstrated a significant improvement in PFS with alectinib (a second-generation ALK TKI) compared with crizotinib (25.7 vs. 10.4 months, HR: 0.50, 95%CI: 0.36–0.70, *p* < 0.001) by independent review, and with a better toxicity profile, as first-line treatment in *ALK*-positive NSCLC patients (65). The EMA and FDA approved alectinib as first-line treatment in 12 October 2017 and in 6 November 2017, respectively. Treatment strategies in this population are provided below and in Figure 1.

### Incidence of BM in ALK-Positive NSCLC

In *ALK*-positive NSCLC patients, CNS metastases affect from 24 to 42% of patients (19, 65–68) with risk increasing over time, reaching 58% at 3 years (19). In this population, median OS after development of BM was 49.5 months, with no survival differences detected according the number of BM (single vs. more than one BM) (69), confirming the prolonged survival of *ALK*-positive NSCLC patients with BM. However, the CNS is a common site of progression with crizotinib; in patients with known BM (treated or untreated), the CNS was a site of new lesions or non-target progression in 70% of cases of progression during crizotinib treatment. In patients without BM at the time of crizotinib initiation, 20% subsequently experienced CNS progression (68, 70). It remains controversial whether this increased risk was an expression of the natural *ALK*-rearranged disease course independent of the therapy received, or if, as in *EGFR*-mutant NSCLC patients, it is related to low CSF penetrance of ALK TKIs. Crizotinib is a substrate for the ATP-binding cassette (ABC) drug efflux transporters, P-gp and ABC subfamily G member 2, and has been associated with poor accumulation of the drug in the brain, a CSF-to-plasma ratio of 0.0026 reported in a case study (71). In support of this, ABCB1−/− and ABCG2−/− mice had a 25- to 70-fold higher brain concentration following oral administration of crizotinib compared to wild type (71).

Nonetheless clinical evidence for crizotinib CNS efficacy has been reported. A pooled retrospective analysis of crizotinib efficacy in *ALK*-positive NSCLC patients with BM from the PROFILE 1005 phase II and PROFILE 1007 phase III trials has been reported (68). At baseline, 31% of patients (275 of 888) had asymptomatic BM. Analytic subgroups were stratified according to prior brain radiotherapy (60%) or not. The intracranial disease control rate (DCR) at 12 weeks was similar in these two groups at 62 and 56%, respectively. Of note, previously treated patients demonstrated higher CNS objective RR with crizotinib (33 vs. 18%, respectively), as well as prolongation of the median time to intracranial progression (13.2 vs. 7.0 months, respectively; Table 2) (68). Intracranial efficacy of crizotinib in treatment-naïve *ALK*-positive patients was studied in the PROFILE 1014 trial (70). Of 343 patients, 79 (23%) had treated BM at baseline. Compared to chemotherapy, crizotinib demonstrated longer PFS (9.0 vs. 4.0 months; HR 0.40, 95% CI: 0.23–0.69, *p* < 0.001) and a better RR (77% vs. 28%, *p* < 0.01). Crizotinib achieved a 12-week intracranial DCR of 85% and median intracranial time to progression of 15.7 months in patients with treated BM (Table 2). CNS progression as the only site of progression on crizotinib was reported in 38% of patients with treated BM at baseline and 19% without (70). In the randomized phase III ALEX trial, crizotinib was used as the standard of care in the control arm and brain MRIs were mandatory at baseline and during follow-up (65). Among the 22 patients with measurable BM at baseline, crizotinib achieved an intracranial RR of 50% and a median duration of intracranial response of 5.5 months. For the seven patients previously treated with brain radiotherapy, crizotinib gave an intracranial RR of 71% and median DOR of 17.3 months (72). Despite these data suggesting intracranial efficacy with crizotinib, especially among previously-treated BM patients, recent data showed that the cumulative incidence rate of CNS progression at 12 months was consistently higher with crizotinib compared with alectinib (32 vs. 4.6% respectively in patients without BM at baseline; HR 0.14, 95% CI: 0.06–0.33, *p* < 0.0001) (65, 72), suggesting that the risk of BM progression may correlate more closely with ALK TKI subtype and not the natural *ALK*-rearranged disease course.

**Table 2:** Efficacy of ALK tyrosine kinase inhibitor (TKIs) in patients with baseline brain metastases (BM).

Drug	Trial (reference)	Brain M1	Measurable Brain M1	icRR (%)	icTTP (months)	s/ic PFS (months)	icDOR (monthss)
Crizotinib	PROFILE 1005 + 1007 pooled. ALK-naïve (previous CT) (68)	275	22/18^b^	18/33^b^	7.0/13.2	NA	26.4,/NR^b^
	PROFILE 1014. Ph III ALK-naïve (70)	79	79	85^c^	15.7	sPFS: 9	NA

Ceritinib	ASCEND 5. Ph III Crizotinib + CT resistant (76)	133	17	35	NA	sPFS: 4.4	6.9
				72.7			16.6
	ASCEND 4. Ph III ALK-naïve (64)	121	22	62	NA	sPFS: 10.7	NA
	ASCEND 3. Ph II ALK TKI-naïve^a^ (75)	49	13	39.4	NA	sPFS: 10.8	9.2
	ASCEND 2. Ph II Crizotinib-resistant (74)	100	33	63	NA	sPFS: 5.4	8.2,
	ASCEND 1. Ph I Naïve and pretreated (73)	94	36	61^d^	NA	NA	11.1^d^

Alectinib	Pooled analysis of ph II. Crizotinib resistant (85)	136	50	64	9.2	NA	10.8
	ALUR ph II. Crizotinib and CT resistant (86, 87)	76	40	54	NA	sPFS: 9.6	17.3
	ALEX. Ph III. ALK TKI-naïve (65, 72)	122	21	81	NA	sPFS: 25.7	

Lorlatinib	Ph I in *ALK*-positive (11% crizotinib-naïve) (90)	41	19	42	NA	sPFS: 9.6	12.4
	Ph I in *ROS1*-positive (90)	12	5	60	NA	sPFS: 7.0	12.0
	Ph II in *ALK/ROS1*-positive (91)						
	ALK TKI treatment-naïve	8	8	75	NA	NR	NA
	Prior crizotinib only and crizotinib ± 1-2 CT	37	37	68	NA	NR	NA
	No-crizotinib TKI ± CT	12	12	42	NA	sPFS: 5.5	NA
	2-3 ALK TKI ± CT	83	83	48	NA	sPFS: 6.9	NA
	*ROS1*-positive any prior line	25	25	56	NA	sPFS:9.6	NA

Brigatinib	Ph I ALK-naïve and crizotinib resistant (93)	46	15	53	NA	icPFS: 15.6	18.9
	ALTA. Ph II in crizotinib-resistant (94, 95)	153	18	67^e^	NA	icPFS: 18.4^e^	NR^e^

*icRR, intracranial response rate; icTTP, intracranial time to progression; s/icPFS, systemic/intracranial progression-free survival (PFS); icDOR, intracranial duration of response; CT, chemotherapy; NA, not available; NR, not reached*.

*^a^ALK TKI naïve and chemotherapy-naïve or up to three lines of chemotherapy with progression during or after the last chemotherapy regimen*.

*^b^Data reported for previously untreated BM/previously treated BM*.

*^c^12-week intracranial disease control rate*.

*^d^Results expressed as ALK inhibitor-naïve patients, ALK inhibitor-pretreated patients*.

*^e^Patients receiving 180 mg/day*.

### Treatment With Novel ALK TKIs

#### Ceritinib

Ceritinib is a second-generation ALK inhibitor that is 20 times as potent as crizotinib. It is effective in *ALK*-positive patients upfront and patients who progress while on crizotinib, including patients with BM (73). In the phase III ASCEND-4 trial, among 121 *ALK*-positive TKI-naïve NSCLC patients with BM, first-line treatment with ceritinib improved PFS (10.7 vs. 6.7 months, HR 0.70) compared to chemotherapy. Intracranial RR in 22 patients with measurable BM at baseline was 73%, with a median duration of intracranial response of 16.6 months (64). The CNS efficacy of ceritinib in crizotinib-pretreated and ALK-naïve patients was tested in the phase I ASCEND-1 trial (73), as well as the phase II ASCEND-2 (74), and ASCEND-3 (75) trials (Table 2). In the phase III, ASCEND-5 trial in previously treated (chemotherapy and crizotinib) *ALK*-positive NSCLC patients, ceritinib compared with chemotherapy significantly improved PFS across all patient subgroups, including in 133 patients with BM at baseline (56% previously treated with brain radiotherapy), from 1.5 months to 4.4 months (HR 0.54, 95% CI: 0.36–0.80). Among the 17 patients with measurable BM, ceritinib gave a 35% intracranial RR and median duration of intracranial response of 6.9 months (76). Nonetheless, despite these second-generation more potent ALK TKIs, BM remained the main site of progression among patients with BM at baseline (76). Based on ceritinib efficacy, an international prospective phase II open-label study is ongoing (ASCEND-7, NCT02336451) specifically evaluating the anti-tumor activity of ceritinib in *ALK*-positive NSCLC patients with BM or leptomeningeal disease (previously treated with radiotherapy or not).

Gastrointestinal toxicity by ceritinib may reduce treatment compliance. The ASCEND-8 (NCT02299505) aimed to evaluate whether administering ceritinib, 450 or 600 mg, with a low-fat meal may enhance gastrointestinal tolerability vs. 750 mg fasted while maintaining similar exposure in 267 treatment-naive ALK-positive NSCLC (neurologically stable BM were stable). The study demonstrated similar efficacy in terms of ORR and DCR with less frequent dose reductions/interruptions and higher relative dose intensity. Ceritinib administered at 450 mg fed dose demonstrated an ORR of 78% and a median PFS of 17.6 months, suggesting this dose as a potential new treatment regimen. However, fed doses of ceritinib in patients with BM were not reported to provide a clear recommendation in this subset (77, 78).

#### Alectinib

Alectinib is a potent ALK TKI, active against several *ALK* mutations that confer resistance to crizotinib (79). It is able to penetrate the CNS and activity is expected based on animal models showing high brain-to-plasma ratios (0.63–0.94) and activity in intracranial tumor implantation models. Unlike crizotinib and ceritinib, preclinical studies suggest that alectinib is not a substrate of P-gp, a key drug efflux pump typically expressed in the BBB, and that it has greater CNS activity than other ALK TKIs (80). In the clinic, alectinib gave an intracranial RR of 52% in 21 crizotinib-resistant patients with baseline BM treated in a phase I trial (81). Alectinib was approved by the FDA in 2015 for *ALK*-positive crizotinib-resistant NSCLC patients based on two phase II clinical trials demonstrating a systemic objective RR of 50–52% (82, 83). A pooled analysis evaluating systemic efficacy of alectinib in both phase II trials enrolling 225 *ALK*-positive crizotinib-resistant NSCLC patients has been performed. Alectinib gave a systemic RR of 51%, and median PFS and OS of 8.3 and 26 months, respectively (84), with 11% of patients having CNS as the only site of progression (85). Intracranial efficacy of alectinib in this population was assessed in 136 crizotinib-resistant patients with BM (37% with measurable disease and 70% previously treated). Intracranial RR in the whole population was 43% (36% in previously irradiated vs. 59% in patients without prior radiation) with a median DOR of 11.1 months. For patients with measurable disease (*N* = 50), the intracranial RR was 64%, with complete response in 22%, and median intracranial DOR was 10.8 months (85). The phase III ALUR trial comparing alectinib with chemotherapy in 107 previously treated (chemotherapy and crizotinib) *ALK*-positive NSCLC patients, reported improved outcome with alectinib (PFS 9.6 vs. 1.4 months, HR 0.15; 95% CI: 0.08–0.29; *p* < 0.001) (86). Among 76 patients with baseline BM, alectinib achieved an intracranial RR of 36% reaching to 54% among the 40 patients with measurable BM (87). These results endorse preclinical data showing promising CNS efficacy profile with alectinib.

In the phase III ALEX trial, 303 previously untreated *ALK*-positive (by immunohistochemistry) NSCLC patients were randomized to receive either alectinib (600 mg twice daily) or crizotinib (250 mg twice daily). Crossover was not allowed. As mentioned previously, PFS was significantly longer with alectinib than with crizotinib and delayed the onset of BM (65). In the ALEX trial, 122 out of 303 (40%) patients had asymptomatic BM at baseline. Alectinib achieved an intracranial RR of 59% with a systemic PFS similar to that reported in the whole population (HR 0.40, 95% CI: 0.25–0.64, *p* < 0.0001) (65), with a median PFS of 14 months among patients with BM at baseline who had not received previous radiotherapy (72). Among the 21 patients with measurable BM, alectinib gave an intracranial RR of 81% and median DOR of 17.3 months (65) (Table 2). Patients with previously-irradiated BM measurable lesions had higher intracranial RR (86 vs. 79%) and intracranial DOR (not reached vs. 17.3 months), compared with patients without prior radiotherapy (72). Similarly, the phase III J-ALEX trial in 207 Japanese *ALK*-positive NSCLC patients demonstrated the superiority of alectinib in terms of PFS over crizotinib (HR 0.34, 95% CI: 0.17–0.71, *p* < 0.0001), and delayed risk of CNS progression in patients with BM at baseline (HR 0.16, 95% CI: 0.02–1.28) and those without (HR 0.41, 95% CI: 0.17–1.01). Among 43 patients with BM at baseline, alectinib significantly improved systemic PFS over crizotinib as first-line treatment (HR 0.08; 95% CI: 0.01–0.61) (88).

#### Lorlatinib

Lorlatinib (PF06463922) is a selective, potent, brain-penetrant next-generation ALK and ROS1 TKI, active against most known resistance mutations (79, 89). Lorlatinib was tested in a phase I trial in 54 pretreated or treatment-naïve *ALK*- (*N* = 41) or *ROS-1* (*N* = 12) positive NSCLC patients (11% treatment-naïve, 52% two or more previous TKIs, and 72% with BM). Patients reached a RR of 46% in the *ALK*-positive population irrespective of the number of prior ALK TKI therapies, and median PFS and DOR of 9.6 and 12.4 months, respectively. Lorlatinib was highly active in the CNS, including intracranial responses in 8 of 19 (42%) *ALK*-positive patients with baseline measurable BM, in over a half of whom two or more previous ALK TKIs had failed (90) (Table 2). The recommended dose for the phase II trial was 100 mg/day.

In the phase II trial (91), lorlatinib conferred a clinically meaningful benefit, including substantial intracranial efficacy ranging from 42 to 75% in patients with advanced *ALK*-positive disease who were treatment-naïve or who had received a range of prior ALK inhibitors and/or chemotherapies (Table 2). In the treatment-naïve cohort (*N* = 30), lorlatinib achieved an RR of 90%, neither PFS nor DOR were reached, while the intracranial RR was 75% among eight patients with BM at baseline. Among the 111 heavily pretreated (two or three previous TKI with or without chemotherapy) patients, lorlatinib reached an overall RR of 39% with median PFS of 6.9 months, and 48% intracranial response among 48 patients with BM at baseline (91). Lorlatinib received breakthrough therapy designation in April 2017 for *ALK*-positive patients previously treated with at least one ALK TKI. Based on these results, the ongoing randomized phase III CROWN trial (NCT03052608) is assessing the efficacy of lorlatinib compared to crizotinib as first-line treatment in *ALK*-positive NSCLC patients. Asymptomatic and pretreated BM are not exclusion criteria.

#### Brigatinib

Brigatinib is another new ALK TKI (also active against *ROS1, EGFR-T790M, IGFR*, and *FLT3* mutations) with a broader spectrum of preclinical activity than ceritinib and alectinib against known crizotinib-resistant *ALK*-mutants (79, 92). Brigatinib was granted break-through therapy designation by the FDA in October 2014 on the basis of its early phase I/II trial data (93). In the phase I trial, among 71 crizotinib-resistant *ALK*-positive NSCLC patients treated with brigatinib the confirmed RR was 62% with a median PFS of 13.2 months. Among 46 patients with BM at baseline, the RR was 53% and 35% for those with measurable (*n* = 15) and non-measurable (*n* = 31) intracranial metastases, respectively. The median intracranial PFS and DOR in this population was 15.6 and 18.9 months, respectively. The recommended dose for the phase II study was determined to be 180 mg/day with a 7-day lead-in at 90 mg to reduce the risk of pulmonary toxicity (93).

In the phase II trial, 222 crizotinib-refractory *ALK*-positive NSCLC patients were randomized to brigatinib 90 mg/day (arm A) or 180 mg/day with a 7-day lead-in at 90 mg (arm B) (94), and updated results were recently presented (95). By independent review, the RR was 51 and 55%, in arms A and B, respectively, and PFS was 9.2 and 16.7 months, respectively, while OS was not reached in arm A and was 27.6 months in arm B. This is the longest PFS in crizotinib-resistant tumors reported with new ALK TKIs. Based on these results, the FDA approved brigatinib in crizotinib-pretreated patients in 28 April 2017. Among the 154 patients with BM at baseline (69%), intracranial RR (by independent-review) in patients with measurable disease (*N* = 44) was 50 and 67% in arm A and B, respectively. For patients with active BM (*N* = 34) RRs were similar to those with baseline BM, 47 and 73% in arms A and B, respectively. Median intracranial PFS was 12.8 and 18.4 months in arms A and B, respectively (95). The intracranial efficacy of brigatinib compares favorably with other second-generation ALK TKIs (74, 85) (Table 2). Brigatinib is currently being investigated in a randomized phase III ALTA-1L (NCT02737501) trial comparing brigatinib vs. crizotinib in *ALK*-positive TKI-naïve patients. Asymptomatic and pretreated BM are not exclusion criteria. This trial allows crossover from crizotinib to brigatinib and may help to elucidate whether a sequential strategy is better than upfront brigatinib.

#### Ensartinib

Efficacy of ensartinib (X-396) 225 mg/day in an expansion study has been reported. Forty of the 80 enrolled patients were evaluable for response, achieving 58% partial responses (88% in eight crizotinib-naïve patients, and 64% in 22 crizotinib-resistant) (96). Updated results among 15 TKI-naïve patients showed an 80% RR and median PFS of 23.8 months (97). CNS responses [(60% partial responses) were observed in both crizotinib-naïve and crizotinib-resistant populations, with a median DOR of 5.8 months (98)]. The ongoing phase III XALT3 (NCT02767804) will compare ensartinib with crizotinib as first-line treatment (previous chemotherapy allowed).

## Other Molecular Alterations: ROS1, RET, BRAF, and NTRK

### ROS1 Rearrangements

*ROS1* rearrangement occurs in approximately 1 to 2% of NSCLC patients. Compared with ALK rearrangements, ROS1 rearrangements are associated with lower rates of extrathoracic metastases, including fewer BM at initial metastatic diagnosis (19 vs. 39%, *p* = 0.033) (99), however *ROS1* does increase the likelihood of BM (100).

In 50 *ROS1-*positive NSCLC patients, crizotinib achieved an RR of 72% and median PFS of 19.2 months (101). Based on these results, the FDA and EMA approved crizotinib for treatment of *ROS1*-positive NSCLC patients in March and August 2016, respectively. Recently, a phase II trial in 32 Asian *ROS1*-positive NSCLC patients, ceritinib gave an RR of 62%, median PFS of 9.3 months (19.3 months among 30 crizotinib-naïve patients), and median OS of 24 months. Among eight patients with BM, intracranial RR with ceritinib was 63% (102). In a phase I trial with lorlatinib, 12 *ROS1*-positive NSCLC patients achieved an intracranial RR of 50% (80% among five patients with target lesions) and median systemic PFS and DOR of 7 and 12 months, respectively (90) (Table 2).

In a phase II study in 47 *ROS*-positive NSCLC patients (28% TKI-naïve, 64% one previous TKI and 8% two or more previous TKIs) treated with lorlatinib, the RR was 36%, with a 45% DCR at 24 weeks, and median PFS and DOR of 9.6 and 13.8 months, respectively (91). Among the 25 patients with BM at baseline, intracranial RR was 56%.

Entrectinib is another ROS1 TKI (also active against *ALK* and *NTRK*) specifically designed to cross the BBB. In a phase I/II trial, entrectinib (600 mg QD) achieved a RR of 78% and median PFS of 29.6 months among 32 treatment-naïve *ROS1*-positive NSCLC patients. The intracranial RR was 83% among 11 patients with BM at baseline (103, 104). Pending questions are the best treatment sequential strategy and whether *ROS1*-positive NSCLC patients with BM should be treated upfront with entrectinib. Given the low *ROS*-1 incidence, it is difficult to perform a randomized trial comparing different treatment strategies.

### RET Rearrangements

In NSCLC, *RET* rearrangements occur in 1 to 2% of unselected cases and 16% of NSCLC tumors that lack other oncogenic drivers. They are more common in adenocarcinomas and in never or lighter-smokers (105, 106). *RET*-rearranged NSCLC patients benefit from pemetrexed-based chemotherapy to a comparable extent as *ALK*- and *ROS1*-rearranged patients (107). Multikinase inhibitors, such as cabozantinib (108) and vandetanib (109, 110) in phase II or retrospective studies (105), have limited efficacy, with RR between 18 and 53%, median PFS between 2.3 and 4.5 months (105, 108–110), and median OS of 6.8 months (105). It has been speculated that the type of fusion partner may play a role in determining treatment response (109); however, this was not validated in the retrospective study (105).

Baseline BM incidence in *RET*-rearranged NSCLC is 27%, without differences in age, smoking status or fusion-partner type. Lifetime incidence of BM in *RET*-rearranged NSCLC patients is 49%. In 37 patients treated with multikinase inhibitors with activity against RET, there were no significant differences in median PFS (2.1 vs. 2.1, *p* = 041) or median OS (3.9 vs. 7.0 months, *p* = 0.10) in patients with BM (*N* = 10) vs. without (*N* = 27) (111). In the phase II trial with cabozantinib, baseline untreated BM were present in five patients. Cabozantinib achieved intracranial disease control in two patients with measurable disease (−34 and −1%). Brain progression during TKI treatment may be less common than in other oncogenic alterations. Of 22 patients who discontinued cabozantinib, BM was the cause in only 10% of cases (111). Similarly, intracranial responses have been reported with alectinib, one patient responding after escalating alectinib to 900 mg twice daily (112). The efficacy of alectinib (900–1200 mg/day) as first-line treatment in *RET*-positive NSCLC patients will be assessed in a multi-cohort phase II/III B-FAST trial (NCT03178552). Treated and asymptomatic BM will be allowed. LOXO-292, another RET TKI has reported tolerability and efficacy in *RET*-dependent cancers even in progressive BM after alectinib (113).

In a phase I trial, vandetanib and everolimus showed anti-tumor activity in *RET*-positive NSCLC patients with BM (114, 115). The short-term outcomes with multikinase inhibitors with activity against RET compared to EGFR/ALK TKIs in *EGFR*-mutant/*ALK*-rearranged NSCLC, strongly suggest that there is a need for more selective and potent RET targeted agents as monotherapy or in combination in order to enhance activity (116).

### BRAF-Mutants

The combination of the BRAF inhibitor dabrafenib with the MEK inhibitor trametinib was approved by the FDA and EMA based on clinical activity in 57 pretreated *BRAF-V600E*-mutated NSCLC patients (1–2% of lung adenocarcinoma patients) following a phase II trial giving an RR of 67% and median PFS and OS of 8.6 and 18.2 months, respectively, however no data regarding CNS efficacy are available (117). Similar outcomes were recently replicated among 36 TKI-naïve BRAF (*V600E*)-mutant NSCLC patients (118). In melanoma BM patients, this combination has reported intracranial responses (119), making it highly probable that activity will be observed in NSCLC patients, although this needs to be validated.

### NTRK Rearrangements

Fusions involving the genes *NTRK1, NTRK2*, and *NTRK3* are oncogenic drivers. They encode the proteins TRKA, TRKB, and TRKC, respectively, and play roles in neuronal development, cell survival, and cellular proliferation (104). These fusion genes have been detected in a variety of tumors including lung in up to 3% of cases, using different assay (NGS or FISH-based) (120). Entrectinib has reported efficacy in *NTRK*-positive tumors, including NSCLC patients, with a median PFS of 15.6 months (104) and also intracranial activity (104, 120), confirming that entrectinib crosses the BBB. Larotrectinib (LOXO 101) is a pan-TRK TKI. In a phase I clinical trial with 55 *NTRK*-positive solid tumors (five NSCLC patients), larotrectinib achieved an RR of 78% across a wide range of ages and tumor types (121). CNS efficacy of this agent remains unknown.

## Upfront TKIs vs. Upfront Radiotherapy in Oncogene-Addicted NSCLC

In oncogene-addicted NSCLC, TKIs have clearly demonstrated increased CNS efficacy, including with next-generation TKIs, which are more potent than first-generation TKIs. Most data have been generated in *EGFR*- or *ALK*-positive patients, although similar outcomes are expected with other druggable alterations. Nonetheless, alternative treatment options exist in this group such as surgery, WBRT or stereotactic radiosurgery (SRS) (122), and the optimal treatment combination or sequence remains unclear.

### Sequential Strategies

A systematic review and meta-analysis of 12 non-comparative studies in 363 *EGFR*-mutant NSCLC patients with BM, showed evidence that upfront radiotherapy (SRS or WBRT) improved survival outcomes (123). However, this study is based on published data and not on individual patient data limiting its validation. This study also reported that radiotherapy caused more neurological adverse events relative to EGFR TKIs alone. In a retrospective multi-institutional analysis in 351 *EGFR*-mutant TKI-naïve NSCLC patients with BM, median OS for three alternative strategies, SRS followed by an EGFR TKI (*n* = 100), WBRT followed by an EGFR TKI (*n* = 120), or an upfront EGFR TKI (*n* = 131), was 46, 30, and 25 months, respectively (*p* < 0.001) (124). In a multivariate analysis, SRS and WBRT vs. EGFR TKI were associated with improved OS, but not with median time to intracranial progression, suggesting that an upfront EGFR TKI and deferred radiotherapy is associated with inferior OS. SRS followed by EGFR-TKI resulted in the longest OS and allowed patients to avoid the potential neurocognitive sequelae of WBRT. However, the retrospective setting meant that data for quality of life and chronic neurocognitive assessments, extracranial disease burden were unavailable, and randomized study design was not used, all of which can be considered as limitations of this analysis. In addition, it is likely that there were a higher number of oligo-metastatic patients in the SRS arm, in whom there is not an urgent need for a TKI to control the extracranial disease, which would generate a major bias.

In a retrospective study (*n* = 97), intracranial PFS was improved in patients who received upfront radiotherapy followed by icotinib compared to those receiving icotinib alone, although without OS improvement (125). However, the absence of randomization makes it difficult to draw a conclusion. On the other hand, in a phase III trial, upfront icotinib (*N* = 85) compared with WBRT (30 Gy) plus chemotherapy (*N* = 91) in *EGFR*-mutant patients with at least three BM significantly improved intracranial PFS (10.0 vs. 4.8 months; HR 0.56, 95% CI: 0.36–0.90; *p* = 0.014), intracranial RR (67.1 vs. 40.9%, *p* < 0.001), and systemic RR (55.0 vs. 11.1%, *p* < 0.001), with a better toxicity profile. Median OS had no significant difference between the arms (18.0 vs. 20.5 months; HR 0.93, 95% CI: 0.60–1.44, *p* = 0.734) (38).

Any of these studies evaluated radiotherapy strategies compared to third-generation EGFR TKIs, so, a prospective randomized trial evaluating intracranial progression after SRS (to avoid the potential neurocognitive sequelae of WBRT) followed by third-generation EGFR TKI vs. third-generation EGFR TKI followed by SRS is needed. The clinical question has also been raised as to whether SRS as consolidative treatment in brain residual disease after EGFR TKI response could improve intracranial PFS in this population or whether this radiotherapy should be only administered in cases of progression on an EGFR TKI.

### Concomitant Strategies

In a recent meta-analysis, radiotherapy plus EGFR TKIs resulted in a superior RR and DCR, and markedly prolonged the CNS-time to progression and OS of NSCLC patients with BM (126), although patients were not selected according to EGFR status. The role of combining an EGFR TKI with WBRT was investigated in a single arm phase II trial of 40 patients (17 *EGFR*-mutant) (127). Patients received erlotinib 150 mg/day for 1 week, followed by erlotinib with concurrent WBRT (2.5 Gy/day, 5 days per week, to 35 Gy) and underwent formal cognitive testing before enrollment and at each follow-up visit. In the *EGFR*-mutant subset, patients had longer OS compared to wild-type *EGFR* (19.1 vs. 9.3 months, respectively). Erlotinib was well tolerated in combination with WBRT with no unexpected cases of neurotoxicity.

In a retrospective study in 133 *EGFR*-mutant patients with BM, radiotherapy (WBRT, SRS) and EGFR TKIs (erlotinib, gefitinib) improved median cranial PFS (16.0 vs. 11.5 moths, *p* = 0.017) and median OS (22 vs. 15 months, *p* = 0.015) compared with EGFR TKIs alone (128). On the contrary, in another retrospective cohort of 230 *EGFR*-mutant BM NSCLC patients, the addition of WBRT to EGFR TKIs compared to EGFR TKIs alone did not result in significant differences in intracranial PFS (7.4 vs. 6.9, *p* = 0.23) or systemic PFS (7.9 vs. 7.5, *p* = 0.55), and combined treatment was associated with worse survival (26.4 vs. 21.6 months, *p* = 0.049) (129). These results should be interpreted with caution given the sample sizes, absence of evaluation of side effects and non-randomized study design. While it can be argued that EGFR TKIs can be safely administered with concurrent WBRT (although for *ALK*-positive patients no data are available), high level evidence to support this is lacking, and concomitant strategies are not overtly recommended in either clinical guidelines (122) or in a recent systematic review (130). In cases of asymptomatic BM patients, given the unclear potential synergistic cognitive toxicities caused by combined therapies, WBRT or SRS should be delayed when other effective systemic therapies are available. A recent systematic literature review about results of combined irradiation and targeted therapies has been also recently published (131).

### Continuing TKIs With and Without Local Therapy

Many strategies to treat CNS disease in *ALK*-positive NSCLC patients have been reported as case reports, such as high-dose crizotinib with a limited intracranial PFS of 1 month (132) and high-dose pemetrexed in combination with high-dose crizotinib with overall stable cerebral disease for 7 months. However, it remains unknown whether the response is attributable to one or both drugs given at high dose (133). In preclinical models, for enhancing CNS drug penetration, P-gp inhibitors such as elacridar, increased the intracranial concentration of crizotinib ~70-fold (134).

In 120 *ALK*-positive NSCLC patients continuing crizotinib beyond initial progression (51% with brain progression), longer median survival was reported compared with patients who received other chemotherapy (16.4 vs. 5.4 months) (135), although this benefit could also be related to local therapies and more indolent disease in the crizotinib arm. Treating isolated CNS progression with local therapies (surgery and/or radiotherapy) while continuing crizotinib could be viewed as an acceptable option (136). In the PROFILE 1014 study, among 25 patients with intracranial progression on crizotinib, 19 received radiotherapy, while continued crizotinib achieved a median treatment time beyond progression of 5.1 months, which was longer than the 2.9 months achieved with crizotinib beyond progression among patients with extracranial progression (70). In a retrospective single-institution study, local therapy (either surgery or radiotherapy) for BM in *EGFR*-mutant (17 treated with erlotinib) or *ALK*-rearranged (38 treated with crizotinib) NSCLC patients and CNS progression allowed continuation of therapy for an additional 7.1 months (137).

Recent studies have reported that *ALK*-positive NSCLC patients with BM treated with SRS and/or WBRT and TKIs have prolonged survival (68, 69, 138). Given the extended OS for *ALK*-positive patients and frequent need for repeated courses of CNS radiotherapy, SRS is the preferred strategy for minimizing cerebral toxicity. Synergistic efficacy of crizotinib and radiotherapy could be explained by increased BBB permeability and decreased P-gp expression following irradiation (139). These results suggest intracranial interventions and TKIs beyond progression are of value in patients with asymptomatic and limited CNS progression on a TKI. This SRS strategy is being validated in an ongoing phase II clinical trial (NCT02314364) among oncogene-addicted *(EGFR-, ALK-, ROS1-*positive*)* NSCLC patients with up to four BM.

The promising CNS activity of the next-generation TKIs suggests that switching targeted agents may be a reasonable alternative to local therapies. However, prospective data are needed to determine which strategy offers the best OS, intracranial control rate, quality of life and therapeutic ratio, taking into account the number of BM and whether patients are symptomatic at the time of progression.

### Second- or Third-Generation TKIs Upfront or Sequentially

In *EGFR*-mutant NSCLC patients, osimertinib has reported higher intracranial activity compared with chemotherapy (51) and first-generation EGFR TKIs (17), and longer delay of onset of BM (51). However, lack of stratification according the presence of BM, no reported survival benefit with osimertinib and no prospective validation of this efficacy, limit interpretation. Nonetheless, preclinical data strongly support the increased intracranial efficacy of osimertinib compared with other EGFR TKIs (44). The ongoing phase II APPLE trial (NCT02856893) assesses the optimal strategy for delivering osimertinib in *EGFR*-mutant NSCLC patients and will prospectively validate the efficacy of osimertinib among those patients with BM at baseline (stratification criteria and brain MRI will be performed at baseline) and also the time to radiological brain progression respect to with first-generation EGFR TKI (gefitinib) (140).

In *ALK*-positive NSCLC patients, based on this significant PFS improvement with alectinib and the delay of CNS progression compared with the current standard first-line crizotinib in *ALK*-positive NSCLC patients, alectinib has became a new standard treatment, and is approved by the EMA and FDA. However, it has not yet been demonstrated whether upfront treatment with second-generation ALK TKIs impact OS compared with sequential treatment strategies (Figure 1). In *ALK*-positive NSCLC patients, 4-year OS was 57% with upfront crizotinib in the randomized phase III PROFILE 1014 trial (*N* = 172), and was 70% with alectinib (300 mg twice daily) among 43 Japanese patients included in a phase II trial (141). Although data are immature, no survival benefit has been reported with upfront alectinib compared with crizotinib in the ALEX trial (65). Also, in PROFILE 1014, patients who received crizotinib followed by another ALK-TKI had longer OS compared with those randomized to chemotherapy followed by no ALK-TKI or other treatment (who had the poorest OS), suggesting a potential benefit of sequential strategies (142).

In a multicentre retrospective study, OS in patients treated with crizotinib followed by alectinib tended to be longer than in patients treated with alectinib alone (143) and median OS up to 50 months has been reported in patients who receive sequential strategies with upfront crizotinib (144, 145). A French nationwide retrospective cohort (CLINALK study) with 318 *ALK*-positive NSCLC patients reported that patients who received next-generation ALK TKIs after crizotinib progression (ceritinib, alectinib, lorlatinib; *N* = 84) had improved OS, reaching a median of 89.6 months (146). Large-scale prospective studies are needed to confirm these preliminary observations.

Finally, each ALK TKI is associated with a distinct spectrum of *ALK*-resistant mutations, and the frequency of mutations increases significantly after treatment with second-generation ALK TKIs (20% with crizotinib vs. 53% with alectinib) (79). It is important to note that there are few new ALK TKIs that may overcome alectinib resistance, and efficacy is dependent on the acquired *ALK* mutation subtype upon progression on alectinib (79). Lack of a tissue biopsy for molecular profiling at progression and limited access to new ALK TKIs worldwide might limit access to subsequent therapies in alectinib-resistant diseases. Validation of liquid biopsies for dynamic markers of TKI efficacy (147) as well as predictive markers for personalized treatment at progression on ALK TKIs is also a challenge. On the other hand, the high CNS response and the delay in the onset of BM with alectinib, which could have a positive impact on patients’ quality of life, might justify first-line treatment with alectinib in this population.

## Conclusion

Brain metastases are common in NSCLC including in molecularly selected populations, and are associated with a reduced quality of life. A multidisciplinary approach is the optimal strategy in oncogene-addicted NSCLC patients with BM. Based on the available clinical data and long OS in patients with asymptomatic synchronous BM at diagnosis, upfront treatment with TKIs alone should be considered with close CNS surveillance for early intervention in patients with an inadequate CNS response. This strategy may defer CNS radiotherapy and avoid long-term neurologic sequelae associated with local therapies. For patients with symptomatic BM, initial TKI therapy is an option, especially in *EGFR*-mutant and *ALK*-positive NSCLC patients treated with new *EGFR* and ALK TKIs based on their higher CNS efficacy. In other cases, sequential treatment initiated with local therapy followed by a TKI is appropriate. For patients who experience CNS progression with controlled extracranial disease while on TKI treatment, local therapy (preferably SRS) followed by the same TKI is an option in patients with a limited number of lesions or who are asymptomatic. In cases of multiple CNS progression, a switch to another TKI with higher CNS-penetration activity with or without WBRT is appropriate.

## Author Contributions

Review concept, design, acquisition, analysis, and interpretation of data performed equally by BB and JR.

## Conflict of Interest Statement

The authors declare that the research was conducted in the absence of any commercial or financial relationships that could be construed as a potential conflict of interest.

## Footnotes

^1^Available from: www.seer.cancer.gov (Accessed: March 23, 2018).

^2^Available from: http://www.fda.gov/MedicalDevices/ProductsandMedicalProcedures/InVitroDiagnostics/ucm301431.htm (Accessed: March 23, 2018).

^3^Available from: http://www.ema.europa.eu/docs/en_GB/document_library/EPAR_-_Product_Information/human/004124/WC500202022.pdf (Accessed: March 23, 2018).

## References

[1] Ferlay J Soerjomataram I Dikshit R Eser S Mathers C Rebelo M Cancer incidence and mortality worldwide: sources, methods and major patterns in GLOBOCAN 2012. Int J Cancer (2015) 136:E359–86. 10.1002/ijc.29210 25220842

[2] Berghoff AS Schur S Füreder LM Gatterbauer B Dieckmann K Widhalm G Descriptive statistical analysis of a real life cohort of 2419 patients with brain metastases of solid cancers. ESMO Open (2016) 1:e000024. 10.1136/esmoopen-2015-000024 27843591 PMC5070252

[3] Eichler AF Chung E Kodack DP Loeffler JS Fukumura D Jain RK. The biology of brain metastases-translation to new therapies. Nat Rev Clin Oncol (2011) 8:344–56. 10.1038/nrclinonc.2011.58 21487419 PMC3259742

[4] Sperduto PW Kased N Roberge D Xu Z Shanley R Luo X Summary report on the graded prognostic assessment: an accurate and facile diagnosis-specific tool to estimate survival for patients with brain metastases. J Clin Oncol (2012) 30:419–25. 10.1200/JCO.2011.38.0527 22203767 PMC3269967

[5] Peters S Bexelius C Munk V Leighl N. The impact of brain metastasis on quality of life, resource utilization and survival in patients with non-small-cell lung cancer. Cancer Treat Rev (2016) 45:139–62. 10.1016/j.ctrv.2016.03.009 27019457

[6] Barlesi F Mazieres J Merlio J-P Debieuvre D Mosser J Lena H Routine molecular profiling of patients with advanced non-small-cell lung cancer: results of a 1-year nationwide programme of the French cooperative thoracic intergroup (IFCT). Lancet (2016) 387:1415–26. 10.1016/S0140-6736(16)00004-0 26777916

[7] Kris MG Johnson BE Berry LD Kwiatkowski DJ Iafrate AJ Wistuba II Using multiplexed assays of oncogenic drivers in lung cancers to select targeted drugs. JAMA (2014) 311:1998–2006. 10.1001/jama.2014.3741 24846037 PMC4163053

[8] Sperduto PW Yang TJ Beal K Pan H Brown PD Bangdiwala A Estimating survival in patients with lung cancer and brain metastases: an update of the graded prognostic assessment for lung cancer using molecular markers (lung-molGPA). JAMA Oncol (2017) 3(6):827–31. 10.1001/jamaoncol.2016.3834 27892978 PMC5824323

[9] Brastianos PK Carter SL Santagata S Cahill DP Taylor-Weiner A Jones RT Genomic characterization of brain metastases reveals branched evolution and potential therapeutic targets. Cancer Discov (2015) 5:1164–77. 10.1158/2159-8290.CD-15-0369 26410082 PMC4916970

[10] Gandhi L Ignatius Ou S-H Shaw AT Barlesi F Dingemans A-MC Kim D-W Efficacy of alectinib in central nervous system metastases in crizotinib-resistant ALK-positive non-small-cell lung cancer: comparison of RECIST 1.1 and RANO-HGG criteria. Eur J Cancer (1990) 2017(82):27–33. 10.1016/j.ejca.2017.05.019 28646771

[11] Ou S-HI Klempner SJ Azada MC Rausei-Mills V Duma C. Radiation necrosis presenting as pseudoprogression (PsP) during alectinib treatment of previously radiated brain metastases in ALK-positive NSCLC: implications for disease assessment and management. Lung Cancer (2015) 88:355–9. 10.1016/j.lungcan.2015.03.022 25882777

[12] Chuang M-T Liu Y-S Tsai Y-S Chen Y-C Wang C-K. Differentiating radiation-induced necrosis from recurrent brain tumor using MR perfusion and spectroscopy: a meta-analysis. PLoS One (2016) 11:e0141438. 10.1371/journal.pone.0141438 26741961 PMC4712150

[13] Di L Rong H Feng B. Demystifying brain penetration in central nervous system drug discovery. Miniperspective. J Med Chem (2013) 56:2–12. 10.1021/jm301297f 23075026

[14] Pardridge WM. The blood-brain barrier: bottleneck in brain drug development. NeuroRx (2005) 2:3–14. 10.1602/neurorx.2.1.3 15717053 PMC539316

[15] Midha A Dearden S McCormack R. EGFR mutation incidence in non-small-cell lung cancer of adenocarcinoma histology: a systematic review and global map by ethnicity (mutMapII). Am J Cancer Res (2015) 5(9):2892–911. 26609494 PMC4633915

[16] Reguart N Remon J Common EGFR-mutated subgroups (Del19/L858R) in advanced non-small-cell lung cancer: chasing better outcomes with tyrosine-kinase inhibitors. Future Oncol (2015) 11(8):1245–57. 10.2217/fon.15.15 25629371

[17] Soria J-C Ohe Y Vansteenkiste J Reungwetwattana T Chewaskulyong B Lee KH Osimertinib in untreated EGFR-mutated advanced non-small-cell lung cancer. N Engl J Med (2018) 378(2):113–25. 10.1056/NEJMoa1713137 29151359

[18] Iuchi T Shingyoji M Itakura M Yokoi S Moriya Y Tamura H Frequency of brain metastases in non-small-cell lung cancer, and their association with epidermal growth factor receptor mutations. Int J Clin Oncol (2015) 20:674–9. 10.1007/s10147-014-0760-9 25336382

[19] Rangachari D Yamaguchi N VanderLaan PA Folch E Mahadevan A Floyd SR Brain metastases in patients with EGFR-mutated or ALK-rearranged non-small-cell lung cancers. Lung Cancer (2015) 88:108–11. 10.1016/j.lungcan.2015.01.020 25682925 PMC4355240

[20] Hendriks LEL Smit EF Vosse BA Mellema WW Heideman DA Bootsma GP EGFR mutated non-small cell lung cancer patients: more prone to development of bone and brain metastases? Lung Cancer (2014) 84:86–91. 10.1016/j.lungcan.2014.01.006 24529684

[21] Heon S Yeap BY Britt GJ Costa DB Rabin MS Jackman DM Development of central nervous system metastases in patients with advanced non-small cell lung cancer and somatic EGFR mutations treated with gefitinib or erlotinib. Clin Cancer Res (2010) 16:5873–82. 10.1158/1078-0432.CCR-10-1588 21030498 PMC2999638

[22] Patel SH Rimner A Foster A Zhang Z Woo KM Yu HA Patterns of initial and intracranial failure in metastatic EGFR-mutant non-small cell lung cancer treated with erlotinib. Lung Cancer (2017) 108:109–14. 10.1016/j.lungcan.2017.03.010 28625621 PMC5477661

[23] Heon S Yeap BY Lindeman NI Joshi VA Butaney M Britt GJ The impact of initial gefitinib or erlotinib versus chemotherapy on central nervous system progression in advanced non-small cell lung cancer with EGFR mutations. Clin Cancer Res (2012) 18:4406–14. 10.1158/1078-0432.CCR-12-0357 22733536 PMC3682221

[24] Ma X Zhu H Guo H Han A Wang H Jing W Risk factors of brain metastasis during the course of EGFR-TKIs therapy for patients with EGFR-mutated advanced lung adenocarcinoma. Oncotarget (2016) 7:81906–17. 10.18632/oncotarget.11918 27626317 PMC5348441

[25] Matsumoto S Takahashi K Iwakawa R Matsuno Y Nakanishi Y Kohno T Frequent EGFR mutations in brain metastases of lung adenocarcinoma. Int J Cancer (2006) 119:1491–4. 10.1002/ijc.21940 16642476

[26] Omuro AMP Kris MG Miller VA Franceschi E Shah N Milton DT High incidence of disease recurrence in the brain and leptomeninges in patients with nonsmall cell lung carcinoma after response to gefitinib. Cancer (2005) 103:2344–8. 10.1002/cncr.21033 15844174

[27] Lee YJ Choi HJ Kim SK Chang J Moon JW Park IK Frequent central nervous system failure after clinical benefit with epidermal growth factor receptor tyrosine kinase inhibitors in Korean patients with nonsmall-cell lung cancer. Cancer (2010) 116:1336–43. 10.1002/cncr.24877 20066717

[28] Ahn M-J Kim D-W Cho BC Kim S-W Lee JS Ahn J-S Activity and safety of AZD3759 in EGFR-mutant non-small-cell lung cancer with CNS metastases (BLOOM): a phase 1, open-label, dose-escalation and dose-expansion study. Lancet Respir Med (2017) 5(11):891–902. 10.1016/S2213-2600(17)30378-8 29056570

[29] Porta R Sánchez-Torres JM Paz-Ares L Massutí B Reguart N Mayo C Brain metastases from lung cancer responding to erlotinib: the importance of EGFR mutation. Eur Respir J (2011) 37:624–31. 10.1183/09031936.00195609 20595147

[30] Wu Y-L Zhou C Cheng Y Lu S Chen G-Y Huang C Erlotinib as second-line treatment in patients with advanced non-small-cell lung cancer and asymptomatic brain metastases: a phase II study (CTONG-0803). Ann Oncol (2013) 24:993–9. 10.1093/annonc/mds529 23129122

[31] Park SJ Kim HT Lee DH Kim KP Kim S-W Suh C Efficacy of epidermal growth factor receptor tyrosine kinase inhibitors for brain metastasis in non-small cell lung cancer patients harboring either exon 19 or 21 mutation. Lung Cancer (2012) 77:556–60. 10.1016/j.lungcan.2012.05.092 22677429

[32] Iuchi T Shingyoji M Sakaida T Hatano K Nagano O Itakura M Phase II trial of gefitinib alone without radiation therapy for Japanese patients with brain metastases from EGFR-mutant lung adenocarcinoma. Lung Cancer (2013) 82:282–7. 10.1016/j.lungcan.2013.08.016 24021541

[33] Zhang Q Zhang X Yan H Jiang B Xu C Yang J Effects of epidermal growth factor receptor-tyrosine kinase inhibitors alone on EGFR-mutant non-small cell lung cancer with brain metastasis. Thorac Cancer (2016) 7:648–54. 10.1111/1759-7714.12379 27755835 PMC5093172

[34] Hotta K Kiura K Ueoka H Tabata M Fujiwara K Kozuki T Effect of gefitinib (’Iressa’, ZD1839) on brain metastases in patients with advanced non-small-cell lung cancer. Lung Cancer (2004) 46:255–61. 10.1016/j.lungcan.2004.04.036 15474674

[35] Zhang S-R Zhu L-C Jiang Y-P Zhang J Xu R-J Xu Y-S Efficacy of afatinib, an irreversible ErbB family blocker, in the treatment of intracerebral metastases of non-small cell lung cancer in mice. Acta Pharmacol Sin (2017) 38:233–40. 10.1038/aps.2016.107 27840411 PMC5309749

[36] Hoffknecht P Tufman A Wehler T Pelzer T Wiewrodt R Schütz M Efficacy of the irreversible ErbB family blocker afatinib in epidermal growth factor receptor (EGFR) tyrosine kinase inhibitor (TKI)-pretreated non-small-cell lung cancer patients with brain metastases or leptomeningeal disease. J Thorac Oncol (2015) 10:156–63. 10.1097/JTO.0000000000000380 25247337 PMC4276567

[37] Schuler M Wu Y-L Hirsh V O’Byrne K Yamamoto N Mok T First-line afatinib versus chemotherapy in patients with non-small cell lung cancer and common epidermal growth factor receptor gene mutations and brain metastases. J Thorac Oncol (2016) 11:380–90. 10.1016/j.jtho.2015.11.014 26823294

[38] Yang J-J Zhou C Huang Y Feng J Lu S Song Y Icotinib versus whole-brain irradiation in patients with EGFR-mutant non-small-cell lung cancer and multiple brain metastases (BRAIN): a multicentre, phase 3, open-label, parallel, randomised controlled trial. Lancet Respir Med (2017) 5(9):707–16. 10.1016/S2213-2600(17)30262-X 28734822

[39] Yun C-H Mengwasser KE Toms AV Woo MS Greulich H Wong K-K The T790M mutation in EGFR kinase causes drug resistance by increasing the affinity for ATP. Proc Natl Acad Sci U S A (2008) 105:2070–5. 10.1073/pnas.0709662105 18227510 PMC2538882

[40] Sequist LV Waltman BA Dias-Santagata D Digumarthy S Turke AB Fidias P Genotypic and histological evolution of lung cancers acquiring resistance to EGFR inhibitors. Sci Transl Med (2011) 3:75ra26. 10.1126/scitranslmed.3002003 21430269 PMC3132801

[41] Yu HA Arcila ME Rekhtman N Sima CS Zakowski MF Pao W Analysis of tumor specimens at the time of acquired resistance to EGFR-TKI therapy in 155 patients with EGFR-mutant lung cancers. Clin Cancer Res (2013) 19:2240–7. 10.1158/1078-0432.CCR-12-2246 23470965 PMC3630270

[42] Hata A Katakami N Yoshioka H Takeshita J Tanaka K Nanjo S Rebiopsy of non-small cell lung cancer patients with acquired resistance to epidermal growth factor receptor-tyrosine kinase inhibitor: comparison between T790M mutation-positive and mutation-negative populations. Cancer (2013) 119:4325–32. 10.1002/cncr.28364 24105277

[43] Hata A Katakami N Yoshioka H Kaji R Masago K Fujita S Spatiotemporal T790M heterogeneity in individual patients with EGFR-mutant non-small-cell lung cancer after acquired resistance to EGFR-TKI. J Thorac Oncol (2015) 10:1553–9. 10.1097/JTO.0000000000000647 26309190

[44] Ballard P Yates JWT Yang Z Kim D-W Yang JC-H Cantarini M Preclinical Comparison of osimertinib with other EGFR-TKIs in EGFR-mutant NSCLC brain metastases models, and early evidence of clinical brain metastases activity. Clin Cancer Res (2016) 22:5130–40. 10.1158/1078-0432.CCR-16-0399 27435396

[45] Yang JC-H Ahn M-J Kim D-W Ramalingam SS Sequist LV Su W-C Osimertinib in pretreated T790M-positive advanced non-small-cell lung cancer: AURA study phase II extension component. J Clin Oncol (2017) 35(12):1288–96. 10.1200/JCO.2016.70.3223 28221867

[46] Goss G Tsai C-M Shepherd FA Bazhenova L Lee JS Chang G-C Osimertinib for pretreated EGFR Thr790Met-positive advanced non-small-cell lung cancer (AURA2): a multicentre, open-label, single-arm, phase 2 study. Lancet Oncol (2016) 17:1643–52. 10.1016/S1470-2045(16)30508-3 27751847

[47] Mok TS Wu Y-L Ahn M-J Garassino MC Kim HR Ramalingam SS Osimertinib or platinum-pemetrexed in EGFR T790M-positive lung cancer. N Engl J Med (2017) 376(7):629–40. 10.1056/NEJMoa1612674 27959700 PMC6762027

[48] Yang J Ramalingam SS Jänne PA Cantarini M Mitsudomi T LBA2_PR: osimertinib (AZD9291) in pre-treated pts with T790M-positive advanced NSCLC: updated phase 1 (P1) and pooled phase 2 (P2) results. J Thorac Oncol (2016) 11:S152–3. 10.1016/S1556-0864(16)30325-2

[49] Goss G Tsai C-M Shepherd FA Ahn M-J Bazhenova L Crinò L CNS response to osimertinib in patients with T790M-positive advanced NSCLC: pooled data from two phase II trials. Ann Oncol (2017). 10.1093/annonc/mdx820 29293889

[50] Goss G Tsai C-M Shepherd F Ahn M-J Bazhenova L Crinò L MA16.11 CNS response to osimertinib in patients with T790M-positive advanced NSCLC: pooled data from two phase II trials. J Thorac Oncol (2017) 12:S440–1. 10.1016/j.jtho.2016.11.514 29293889

[51] Mok T Ahn M-J Han J-Y Kang JH Katakami N Kim H CNS response to osimertinib in patients (pts) with T790M-positive advanced NSCLC: data from a randomized phase III trial (AURA3). J Clin Oncol (2017) 35:9005–9005. 10.1200/JCO.2017.35.15_suppl.9005

[52] Zhang J Yu J Sun X Meng X. Epidermal growth factor receptor tyrosine kinase inhibitors in the treatment of central nerve system metastases from non-small cell lung cancer. Cancer Lett (2014) 351:6–12. 10.1016/j.canlet.2014.04.019 24861428

[53] Vansteenkiste J Reungwetwattana T Nakagawa K Cho BC Dols MAC Cho EK LBA5CNS response to osimertinib vs standard of care (SoC) EGFR-TKI as first-line therapy in patients (pts) with EGFR-TKI sensitising mutation (EGFRm)-positive advanced non-small cell lung cancer (NSCLC): data from the FLAURA study. Ann Oncol (2017) 28:.007–.729. 10.1093/annonc/mdx729.007

[54] Jackman DM Holmes AJ Lindeman N Wen PY Kesari S Borras AM Response and resistance in a non-small-cell lung cancer patient with an epidermal growth factor receptor mutation and leptomeningeal metastases treated with high-dose gefitinib. J Clin Oncol (2006) 24:4517–20. 10.1200/JCO.2006.06.6126 16983123

[55] Grommes C Oxnard GR Kris MG Miller VA Pao W Holodny AI “Pulsatile” high-dose weekly erlotinib for CNS metastases from EGFR mutant non-small cell lung cancer. Neuro Oncol (2011) 13:1364–9. 10.1093/neuonc/nor121 21865399 PMC3223088

[56] Kuiper JL Smit EF High-dose, pulsatile erlotinib in two NSCLC patients with leptomeningeal metastases – one with a remarkable thoracic response as well. Lung Cancer (2013) 80:102–5. 10.1016/j.lungcan.2012.12.024 23375403

[57] Kuiper JL Heideman DAM Thunnissen E van Wijk AW Postmus PE Smit EF High-dose, weekly erlotinib is not an effective treatment in EGFR-mutated non-small cell lung cancer-patients with acquired extracranial progressive disease on standard dose erlotinib. Eur J Cancer (1990) 2014(50):1399–401. 10.1016/j.ejca.2014.02.005 24582911

[58] Yu HA Sima C Feldman D Liu LL Vaitheesvaran B Cross J Phase 1 study of twice weekly pulse dose and daily low-dose erlotinib as initial treatment for patients with EGFR-mutant lung cancers†. Ann Oncol (2017) 28:278–84. 10.1093/annonc/mdw556 28073786 PMC5834093

[59] Besse B Le Moulec S Mazieres J Senellart H Barlesi F Chouaid C Bevacizumab in patients with nonsquamous non-small cell lung cancer and asymptomatic, untreated brain metastases (BRAIN): a nonrandomized, phase II study. Clin Cancer Res (2015) 21(8):1896–903. 10.1158/1078-0432.CCR-14-2082 25614446

[60] Ilhan-Mutlu A Osswald M Liao Y Gömmel M Reck M Miles D Bevacizumab prevents brain metastases formation in lung adenocarcinoma. Mol Cancer Ther (2016) 15:702–10. 10.1158/1535-7163.MCT-15-0582 26809491

[61] Seto T Kato T Nishio M Goto K Atagi S Hosomi Y Erlotinib alone or with bevacizumab as first-line therapy in patients with advanced non-squamous non-small-cell lung cancer harbouring EGFR mutations (JO25567): an open-label, randomised, multicentre, phase 2 study. Lancet Oncol (2014) 15:1236–44. 10.1016/S1470-2045(14)70381-X 25175099

[62] Rosell R Dafni U Felip E Curioni-Fontecedro A Gautschi O Peters S Erlotinib and bevacizumab in patients with advanced non-small-cell lung cancer and activating EGFR mutations (BELIEF): an international, multicentre, single-arm, phase 2 trial. Lancet Respir Med (2017) 5:435–44. 10.1016/S2213-2600(17)30129-7 28408243

[63] Novello S Barlesi F Califano R Cufer T Ekman S Levra MG Metastatic non-small-cell lung cancer: ESMO clinical practice guidelines for diagnosis, treatment and follow-up. Ann Oncol (2016) 27:v1–27. 10.1093/annonc/mdw326 27664245

[64] Soria J-C Tan DSW Chiari R Wu Y-L Paz-Ares L Wolf J First-line ceritinib versus platinum-based chemotherapy in advanced ALK-rearranged non-small-cell lung cancer (ASCEND-4): a randomised, open-label, phase 3 study. Lancet (2017) 389:917–29. 10.1016/S0140-6736(17)30123-X 28126333

[65] Peters S Camidge DR Shaw AT Gadgeel S Ahn JS Kim D-W Alectinib versus crizotinib in untreated ALK-positive non-small-cell lung cancer. N Engl J Med (2017) 377(9):829–38. 10.1056/NEJMoa1704795 28586279

[66] Shaw AT Kim D-W Nakagawa K Seto T Crino L Ahn M-J Crizotinib versus chemotherapy in advanced ALK-positive lung cancer. N Engl J Med (2013) 368:2385–94. 10.1056/NEJMoa1214886 23724913

[67] Shaw AT Kim D-W Mehra R Tan DSW Felip E Chow LQM Ceritinib in ALK-rearranged non-small-cell lung cancer. N Engl J Med (2014) 370:1189–97. 10.1056/NEJMoa1311107 24670165 PMC4079055

[68] Costa DB Shaw AT Ou S-HI Solomon BJ Riely GJ Ahn M-J Clinical experience with crizotinib in patients with advanced ALK-rearranged non-small-cell lung cancer and brain metastases. J Clin Oncol (2015) 33:1881–8. 10.1200/JCO.2014.59.0539 25624436 PMC4451171

[69] Johung KL Yeh N Desai NB Williams TM Lautenschlaeger T Arvold ND Extended survival and prognostic factors for patients with ALK-rearranged non-small-cell lung cancer and brain metastasis. J Clin Oncol (2016) 34:123–9. 10.1200/JCO.2015.62.0138 26438117 PMC5070549

[70] Solomon BJ Cappuzzo F Felip E Blackhall FH Costa DB Kim D-W Intracranial efficacy of crizotinib versus chemotherapy in patients with advanced ALK-positive non-small-cell lung cancer: results from PROFILE 1014. J Clin Oncol (2016) 34:2858–65. 10.1200/JCO.2015.63.5888 27022118

[71] Costa DB Kobayashi S Pandya SS Yeo W-L Shen Z Tan W CSF concentration of the anaplastic lymphoma kinase inhibitor crizotinib. J Clin Oncol (2011) 29:e443–5. 10.1200/JCO.2010.34.1313 21422405

[72] Gadgeel S Peters S Mok TSK Shaw AT Kim D-W Ou S-HI 1298O_PRAlectinib vs crizotinib in treatment-naïve ALK+ NSCLC: CNS efficacy results from the ALEX study. Ann Oncol (2017) 28:.057–.440. 10.1093/annonc/mdx440.057 PMC6290889 30215676

[73] Kim D-W Mehra R Tan DSW Felip E Chow LQM Camidge DR Activity and safety of ceritinib in patients with ALK-rearranged non-small-cell lung cancer (ASCEND-1): updated results from the multicentre, open-label, phase 1 trial. Lancet Oncol (2016) 17:452–63. 10.1016/S1470-2045(15)00614-2 26973324 PMC5063047

[74] Crinò L Ahn M-J De Marinis F Groen HJ Wakelee H Hida T Multicenter phase II study of whole-body and intracranial activity with ceritinib in patients with ALK-rearranged non-small-cell lung cancer previously treated with chemotherapy and crizotinib: results from ASCEND-2. J Clin Oncol (2016) 34(24):2866–73. 10.1200/JCO.2015.65.5936 27432917

[75] Felip E Orlov S Park K Yu C-J Tsai C-M Nishio M Phase 2 study of ceritinib in ALKi-naïve patients (pts) with ALK-rearranged (ALK+) non-small cell lung cancer (NSCLC): whole body responses in the overall pt group and in pts with baseline brain metastases (BM). Ann Oncol (2016) 27:1208O 10.1093/annonc/mdw383.03

[76] Shaw AT Kim TM Crinò L Gridelli C Kiura K Liu G Ceritinib versus chemotherapy in patients with ALK-rearranged non-small-cell lung cancer previously given chemotherapy and crizotinib (ASCEND-5): a randomised, controlled, open-label, phase 3 trial. Lancet Oncol (2017) 18:874–86. 10.1016/S1470-2045(17)30339-X 28602779

[77] Cho BC Kim D-W Bearz A Laurie SA McKeage M Borra G ASCEND-8: a randomized phase 1 study of ceritinib, 450 mg or 600 mg, taken with a low-fat meal versus 750 mg in fasted state in patients with anaplastic lymphoma kinase (ALK)-rearranged metastatic non–small cell lung cancer (NSCLC). J Thorac Oncol 12:1357–67. 10.1016/j.jtho.2017.07.005 28729021

[78] Cho BC Obermannová R Bearz A Kim D Orlov S Borra G OA 05.07 efficacy and updated safety of ceritinib (450 Mg or 600 Mg) with low-fat meal vs 750 Mg fasted in ALK+ metastatic NSCLC. J Thorac Oncol 12:S1757 10.1016/j.jtho.2017.09.352

[79] Gainor JF Dardaei L Yoda S Friboulet L Leshchiner I Katayama R Molecular mechanisms of resistance to first- and second-generation ALK inhibitors in ALK-rearranged lung cancer. Cancer Discov (2016) 6:1118–33. 10.1158/2159-8290.CD-16-0596 27432227 PMC5050111

[80] Gainor JF Sherman CA Willoughby K Logan J Kennedy E Brastianos PK Alectinib salvages CNS relapses in ALK-positive lung cancer patients previously treated with crizotinib and ceritinib. J Thorac Oncol (2015) 10:232–6. 10.1097/JTO.0000000000000455 25526238 PMC4304931

[81] Gadgeel SM Gandhi L Riely GJ Chiappori AA West HL Azada MC Safety and activity of alectinib against systemic disease and brain metastases in patients with crizotinib-resistant ALK-rearranged non-small-cell lung cancer (AF-002JG): results from the dose-finding portion of a phase 1/2 study. Lancet Oncol (2014) 15:1119–28. 10.1016/S1470-2045(14)70362-6 25153538

[82] Shaw AT Gandhi L Gadgeel S Riely GJ Cetnar J West H Alectinib in ALK-positive, crizotinib-resistant, non-small-cell lung cancer: a single-group, multicentre, phase 2 trial. Lancet Oncol (2016) 17:234–42. 10.1016/S1470-2045(15)00488-X 26708155 PMC4752892

[83] Ou S-HI Ahn JS De Petris L Govindan R Yang JC-H Hughes B Alectinib in crizotinib-refractory ALK-rearranged non-small-cell lung cancer: a phase II global study. J Clin Oncol (2016) 34:661–8. 10.1200/JCO.2015.63.9443 26598747

[84] Yang JC-H Ou S-I De Petris L Gadgeel S Gandhi L Kim D-W Pooled systemic efficacy and safety data from the pivotal phase II studies (NP28673 and NP28761) of alectinib in ALK-positive non-small-cell lung cancer. J Thorac Oncol (2017) 12(10):1552–60. 10.1016/j.jtho.2017.06.070 28689043 PMC6886236

[85] Gadgeel SM Shaw AT Govindan R Gandhi L Socinski MA Camidge DR Pooled analysis of CNS response to alectinib in two studies of pretreated patients with ALK-positive non-small-cell lung cancer. J Clin Oncol (2016) 34:4079–85. 10.1200/JCO.2016.68.4639 27863201 PMC7845943

[86] Novello S Mazieres J Oh I-J de Castro J Migliorino MR Helland A 1299O_PRPrimary results from the phase III ALUR study of alectinib versus chemotherapy in previously treated ALK+ non-small-cell lung cancer (NSCLC). Ann Oncol (2017) 28:.058–.440. 10.1093/annonc/mdx440.058

[87] de Castro J Novello S Mazieres J Oh I-J Migliorino MR Helland A 1346PCNS efficacy results from the phase III ALUR study of alectinib vs chemotherapy in previously treated ALK+ NSCLC. Ann Oncol (2017) 28:.048–.380. 10.1093/annonc/mdx380.048

[88] Hida T Nokihara H Kondo M Kim YH Azuma K Seto T Alectinib versus crizotinib in patients with ALK-positive non-small-cell lung cancer (J-ALEX): an open-label, randomised phase 3 trial. Lancet (2017) 390:29–39. 10.1016/S0140-6736(17)30565-2 28501140

[89] Zou HY Friboulet L Kodack DP Engstrom LD Li Q West M PF-06463922, an ALK/ROS1 inhibitor, overcomes resistance to first and second generation ALK inhibitors in preclinical models. Cancer Cell (2015) 28:70–81. 10.1016/j.ccell.2015.05.010 26144315 PMC4504786

[90] Shaw AT Felip E Bauer TM Besse B Navarro A Postel-Vinay S Lorlatinib in non-small-cell lung cancer with ALK or ROS1 rearrangement: an international, multicentre, open-label, single-arm first-in-man phase 1 trial. Lancet Oncol (2017) 18(12):1590–9. 10.1016/S1470-2045(17)30680-0 29074098 PMC5777233

[91] Solomon B Shaw A Ou S Besse B Felip E Bauer T OA 05.06 phase 2 study of lorlatinib in patients with advanced ALK+/ROS1+ non-small-cell lung cancer. J Thorac Oncol 12:S1756 10.1016/j.jtho.2017.09.351

[92] Zhang S Anjum R Squillace R Nadworny S Zhou T Keats J The potent ALK inhibitor brigatinib (AP26113) overcomes mechanisms of resistance to first- and second-generation ALK inhibitors in preclinical models. Clin Cancer Res (2016) 22:5527–38. 10.1158/1078-0432.CCR-16-0569 27780853

[93] Gettinger SN Bazhenova LA Langer CJ Salgia R Gold KA Rosell R Activity and safety of brigatinib in ALK-rearranged non-small-cell lung cancer and other malignancies: a single-arm, open-label, phase 1/2 trial. Lancet Oncol (2016) 17:1683–96. 10.1016/S1470-2045(16)30392-8 27836716

[94] Kim D-W Tiseo M Ahn M-J Reckamp KL Hansen KH Kim S-W Brigatinib in patients with crizotinib-refractory anaplastic lymphoma kinase-positive non-small-cell lung cancer: a randomized, multicenter phase II trial. J Clin Oncol (2017) 35(22):2490–8. 10.1200/JCO.2016.71.5904 28475456

[95] Ahn M Camidge DR Tiseo M Reckamp K Hansen K Kim S OA 05.05 brigatinib in crizotinib-refractory ALK+ NSCLC: updated efficacy and safety results from ALTA, a randomized phase 2 trial. J Thorac Oncol (2017) 12:S1755–6. 10.1016/j.jtho.2017.09.350

[96] Horn L Wakelee H Reckamp K Blumenschein G Infante J Carter C P3.02a-001 response and plasma genotyping from phase I/II trial of ensartinib (X-396) in patients (Pts) with ALK+ NSCLC. J Thorac Oncol (2017) 12:S1159 10.1016/j.jtho.2016.11.1631

[97] Wakelee H Sanborn R Nieva J Waqar S Brzezniak C Bauman J MA 07.02 response to ensartinib in TKI naïve ALK+ NSCLC patients. J Thorac Oncol 12:S1826 10.1016/j.jtho.2017.09.504

[98] Reckamp KL Wakelee HA Patel S Blumenschein G Neal JW Gitlitz B 88OCNS activity of ensartinib in ALK+ non-small cell lung cancer (NSCLC) patients (pts). Ann Oncol (2017) 28:mdx091.008 10.1093/annonc/mdx091.008

[99] Gainor JF Tseng D Yoda S Dagogo-Jack I Friboulet L Lin JJ Patterns of metastatic spread and mechanisms of resistance to crizotinib in ROS1-positive non–small-cell lung cancer. JCO Precis Oncol (2017) 1:1–13. 10.1200/PO.17.00063 PMC5766287 29333528

[100] Preusser M Streubel B Birner P ROS1 translocations and amplifications in lung cancer brain metastases. J Neurooncol (2014) 118:425–6. 10.1007/s11060-014-1446-x 24760415

[101] Shaw AT Ou S-HI Bang Y-J Camidge DR Solomon BJ Salgia R Crizotinib in ROS1-rearranged non-small-cell lung cancer. N Engl J Med (2014) 371:1963–71. 10.1056/NEJMoa1406766 25264305 PMC4264527

[102] Lim SM Kim HR Lee J-S Lee KH Lee Y-G Min YJ Open-label, multicenter, phase II study of ceritinib in patients with non-small-cell lung cancer harboring ROS1 rearrangement. J Clin Oncol (2017) 35(23):2613–8. 10.1200/JCO.2016.71.3701 28520527

[103] Ahn M Cho BC Siena S Drilon A De Braud F Krebs M OA 14.06 entrectinib in patients with locally advanced or metastatic ROS1 fusion-positive non-small cell lung cancer (NSCLC). J Thorac Oncol 12:S1783 10.1016/j.jtho.2017.09.411 PMC8078299 33646820

[104] Drilon A Siena S Ou S-HI Patel M Ahn MJ Lee J Safety and antitumor activity of the multitargeted pan-TRK, ROS1, and ALK inhibitor entrectinib: combined results from two phase I trials (ALKA-372-001 and STARTRK-1). Cancer Discov (2017) 7:400–9. 10.1158/2159-8290.CD-16-1237 28183697 PMC5380583

[105] Gautschi O Milia J Filleron T Wolf J Carbone DP Owen D Targeting RET in patients with RET-rearranged lung cancers: results from the global, multicenter ret registry. J Clin Oncol (2017) 35:1403–10. 10.1200/JCO.2016.70.9352 28447912 PMC5559893

[106] Ferrara R Auger N Auclin E Besse B Clinical and translational implications of RET rearrangements in non-small cell lung cancer. J Thorac Oncol (2018) 13(1):27–45. 10.1016/j.jtho.2017.10.021 29128428

[107] Drilon A Bergagnini I Delasos L Sabari J Woo KM Plodkowski A Clinical outcomes with pemetrexed-based systemic therapies in RET-rearranged lung cancers. Ann Oncol (2016) 27:1286–91. 10.1093/annonc/mdw163 27056998 PMC4922319

[108] Drilon A Rekhtman N Arcila M Wang L Ni A Albano M Cabozantinib in patients with advanced RET-rearranged non-small-cell lung cancer: an open-label, single-centre, phase 2, single-arm trial. Lancet Oncol (2016) 17:1653–60. 10.1016/S1470-2045(16)30562-9 27825636 PMC5143197

[109] Yoh K Seto T Satouchi M Nishio M Yamamoto N Murakami H Vandetanib in patients with previously treated RET-rearranged advanced non-small-cell lung cancer (LURET): an open-label, multicentre phase 2 trial. Lancet Respir Med (2017) 5:42–50. 10.1016/S2213-2600(16)30322-8 27825616

[110] Lee S-H Lee J-K Ahn M-J Kim D-W Sun J-M Keam B Vandetanib in pretreated patients with advanced non-small cell lung cancer-harboring RET rearrangement: a phase II clinical trial. Ann Oncol (2017) 28:292–7. 10.1093/annonc/mdw559 27803005

[111] Drilon AE Filleron T Bergagnini I Milia J Hatzoglou V Velcheti V Baseline frequency of brain metastases and outcomes with multikinase inhibitor therapy in patients with RET-rearranged lung cancers. J Clin Oncol (2017) 35:9069–9069. 10.1200/JCO.2017.35.15_suppl.9069 PMC6434708 30017832

[112] Lin JJ Kennedy E Sequist LV Brastianos PK Goodwin KE Stevens S Clinical activity of alectinib in advanced RET-rearranged non-small cell lung cancer. J Thorac Oncol (2016) 11:2027–32. 10.1016/j.jtho.2016.08.126 27544060

[113] Velcheti V Bauer T Subbiah V Cabanillas M Lakhani N Wirth L OA 12.07 LOXO-292, a potent, highly selective RET inhibitor, in MKI-resistant RET fusion-positive lung cancer patients with and without brain metastases. J Thorac Oncol (2017) 12:S1778 10.1016/j.jtho.2017.09.399

[114] Subbiah V Berry J Roxas M Guha-Thakurta N Subbiah IM Ali SM Systemic and CNS activity of the RET inhibitor vandetanib combined with the mTOR inhibitor everolimus in KIF5B-RET re-arranged non-small cell lung cancer with brain metastases. Lung Cancer (2015) 89:76–9. 10.1016/j.lungcan.2015.04.004 25982012 PMC4998046

[115] Cascone T Hess KR Piha-Paul SA Hong DS Subbiah IM Bhatt T Safety, toxicity and activity of multi-kinase inhibitor vandetanib in combination with everolimus in advanced solid tumors. J Clin Oncol (2016) 34:9073–9073. 10.1200/JCO.2016.34.15_suppl.9073

[116] Plenker D Riedel M Brägelmann J Dammert MA Chauhan R Knowles PP Drugging the catalytically inactive state of RET kinase in RET-rearranged tumors. Sci Transl Med (2017) 9:eaah6144. 10.1126/scitranslmed.aah6144 28615362 PMC5805089

[117] Planchard D Besse B Kim TM Quoix EA Souquet PJ Mazieres J Updated survival of patients (pts) with previously treated BRAF V600E–mutant advanced non-small cell lung cancer (NSCLC) who received dabrafenib (D) or D + trametinib (T) in the phase II BRF113928 study. J Clin Oncol (2017) 35:9075–9075. 10.1200/JCO.2017.35.15_suppl.9075

[118] Planchard D Smit EF Groen HJM Mazieres J Besse B Helland Å Dabrafenib plus trametinib in patients with previously untreated BRAF(V600E)-mutant metastatic non-small-cell lung cancer: an open-label, phase 2 trial. Lancet Oncol (2017) 18:1307–16. 10.1016/S1470-2045(17)30679-4 28919011

[119] Davies MA Saiag P Robert C Grob J-J Flaherty KT Arance A Dabrafenib plus trametinib in patients with BRAF(V600)-mutant melanoma brain metastases (COMBI-MB): a multicentre, multicohort, open-label, phase 2 trial. Lancet Oncol (2017) 18:863–73. 10.1016/S1470-2045(17)30429-1 28592387 PMC5991615

[120] Farago AF Le LP Zheng Z Muzikansky A Drilon A Patel M Durable clinical response to entrectinib in NTRK1-rearranged non-small cell lung cancer. J Thorac Oncol (2015) 10:1670–4. 10.1097/01.JTO.0000473485.38553.f0 26565381 PMC4643748

[121] Hyman DM Laetsch TW Kummar S DuBois SG Farago AF Pappo AS The efficacy of larotrectinib (LOXO-101), a selective tropomyosin receptor kinase (TRK) inhibitor, in adult and pediatric TRK fusion cancers. J Clin Oncol (2017) 35:LBA2501–2501. 10.1200/JCO.2017.35.18_suppl.LBA2501

[122] Soffietti R Abacioglu U Baumert B Combs SE Kinhult S Kros JM Diagnosis and treatment of brain metastases from solid tumors: guidelines from the European association of neuro-oncology (EANO). Neuro Oncol (2017) 19:162–74. 10.1093/neuonc/now241 28391295 PMC5620494

[123] Soon YY Leong CN Koh WY Tham IWK. EGFR tyrosine kinase inhibitors versus cranial radiation therapy for EGFR mutant non-small cell lung cancer with brain metastases: a systematic review and meta-analysis. Radiother Oncol (2015) 114:167–72. 10.1016/j.radonc.2014.12.011 25583566

[124] Magnuson WJ Lester-Coll NH Wu AJ Yang TJ Lockney NA Gerber NK Management of brain metastases in tyrosine kinase inhibitor-naïve epidermal growth factor receptor-mutant non-small-cell lung cancer: a retrospective multi-institutional analysis. J Clin Oncol (2017) 35:1070–7. 10.1200/JCO.2016.69.7144 28113019

[125] Fan Y Xu Y Gong L Fang L Lu H Qin J Effects of icotinib with and without radiation therapy on patients with EGFR mutant non-small cell lung cancer and brain metastases. Sci Rep (2017) 7:45193. 10.1038/srep45193 28332624 PMC5362911

[126] Jiang T Min W Li Y Yue Z Wu C Zhou C. Radiotherapy plus EGFR TKIs in non-small cell lung cancer patients with brain metastases: an update meta-analysis. Cancer Med (2016) 5:1055–65. 10.1002/cam4.673 26990668 PMC4924363

[127] Welsh JW Komaki R Amini A Munsell MF Unger W Allen PK Phase II trial of erlotinib plus concurrent whole-brain radiation therapy for patients with brain metastases from non-small-cell lung cancer. J Clin Oncol (2013) 31:895–902. 10.1200/JCO.2011.40.1174 23341526 PMC3577951

[128] Zhu Q Sun Y Cui Y Ye K Yang C Yang D Clinical outcome of tyrosine kinase inhibitors alone or combined with radiotherapy for brain metastases from epidermal growth factor receptor (EGFR) mutant non small cell lung cancer (NSCLC). Oncotarget (2017) 8:13304–11. 10.18632/oncotarget.14515 28076323 PMC5355097

[129] Jiang T Su C Li X Zhao C Zhou F Ren S EGFR TKIs plus WBRT demonstrated no survival benefit other than that of TKIs alone in patients with NSCLC and EGFR mutation and brain metastases. J Thorac Oncol (2016) 11:1718–28. 10.1016/j.jtho.2016.05.013 27237825

[130] Hendriks LEL Schoenmaekers J Zindler JD Eekers DBP Hoeben A De Ruysscher DKM Safety of cranial radiotherapy concurrent with tyrosine kinase inhibitors in non-small cell lung cancer patients: a systematic review. Cancer Treat Rev (2015) 41:634–45. 10.1016/j.ctrv.2015.05.005 25990950

[131] Tallet AV Dhermain F Le Rhun E Noël G Kirova YM. Combined irradiation and targeted therapy or immune checkpoint blockade in brain metastases: toxicities and efficacy. Ann Oncol (2017) 28:2962–76. 10.1093/annonc/mdx408 29045524

[132] Kim YH Ozasa H Nagai H Sakamori Y Yoshida H Yagi Y High-dose crizotinib for brain metastases refractory to standard-dose crizotinib. J Thorac Oncol (2013) 8:e85–6. 10.1097/JTO.0b013e31829cebbb 23945393

[133] Gandhi L Drappatz J Ramaiya NH Otterson GA High-dose pemetrexed in combination with high-dose crizotinib for the treatment of refractory CNS metastases in ALK-rearranged non-small-cell lung cancer. J Thorac Oncol (2013) 8:e3–5. 10.1097/JTO.0b013e3182762d20 23242445

[134] Tang SC Nguyen LN Sparidans RW Wagenaar E Beijnen JH Schinkel AH. Increased oral availability and brain accumulation of the ALK inhibitor crizotinib by coadministration of the P-glycoprotein (ABCB1) and breast cancer resistance protein (ABCG2) inhibitor elacridar. Int J Cancer (2014) 134:1484–94. 10.1002/ijc.28475 24037730

[135] Ou S-HI Jänne PA Bartlett CH Tang Y Kim D-W Otterson GA Clinical benefit of continuing ALK inhibition with crizotinib beyond initial disease progression in patients with advanced ALK-positive NSCLC. Ann Oncol (2014) 25:415–22. 10.1093/annonc/mdt572 24478318

[136] Takeda M Okamoto I Nakagawa K. Clinical impact of continued crizotinib administration after isolated central nervous system progression in patients with lung cancer positive for ALK rearrangement. J Thorac Oncol (2013) 8:654–7. 10.1097/JTO.0b013e31828c28e7 23584297

[137] Weickhardt AJ Scheier B Burke JM Gan G Lu X Bunn PA Local ablative therapy of oligoprogressive disease prolongs disease control by tyrosine kinase inhibitors in oncogene-addicted non-small-cell lung cancer. J Thorac Oncol (2012) 7:1807–14. 10.1097/JTO.0b013e3182745948 23154552 PMC3506112

[138] Mak KS Gainor JF Niemierko A Oh KS Willers H Choi NC Significance of targeted therapy and genetic alterations in EGFR, ALK, or KRAS on survival in patients with non-small cell lung cancer treated with radiotherapy for brain metastases. Neuro Oncol (2015) 17:296–302. 10.1093/neuonc/nou146 25053852 PMC4288518

[139] Sullivan I Planchard D. Treatment modalities for advanced ALK-rearranged non-small-cell lung cancer. Future Oncol (2016) 12:945–61. 10.2217/fon.16.15 26892300

[140] Remon J Menis J Hasan B Peric A De Maio E Novello S The APPLE trial: feasibility and activity of AZD9291 (osimertinib) treatment on positive plasma T790M in EGFR-mutant NSCLC patients. EORTC 1613. Clin Lung Cancer (2017) 18:583–8. 10.1016/j.cllc.2017.02.005 28341106

[141] Nishio M Kiura K Seto T Nakagawa K Maemondo M Inoue A OA 05.08 final result of phase I/II study (AF-001JP) of alectinib, a selective CNS-active ALK inhibitor, in ALK+ NSCLC patients (Pts). J Thorac Oncol 12:S1757 10.1016/j.jtho.2017.09.353

[142] Mok TSK Kim D-W Wu Y-L Nakagawa K Mekhail T Felip E LBA50Overall survival (OS) for first-line crizotinib versus chemotherapy in ALK+ lung cancer: updated results from PROFILE 1014. Ann Oncol (2017) 28:.053–.440. 10.1093/annonc/mdx440.053

[143] Ito K Hataji O Kobayashi H Fujiwara A Yoshida M D’Alessandro-Gabazza CN Sequential therapy with crizotinib and alectinib in ALK-rearranged non-small cell lung cancer-a multicenter retrospective study. J Thorac Oncol (2017) 12:390–6. 10.1016/j.jtho.2016.07.022 27498387

[144] Gainor JF Tan DSW De Pas T Solomon BJ Ahmad A Lazzari C Progression-free and overall survival in ALK-positive NSCLC patients treated with sequential crizotinib and ceritinib. Clin Cancer Res (2015) 21:2745–52. 10.1158/1078-0432.CCR-14-3009 25724526 PMC4470734

[145] Watanabe S Hayashi H Okamoto K Fujiwara K Hasegawa Y Kaneda H Progression-free and overall survival of patients with ALK rearrangement-positive non-small cell lung cancer treated sequentially with crizotinib and alectinib. Clin Lung Cancer (2016) 17:528–34. 10.1016/j.cllc.2016.05.001 27318655

[146] Duruisseaux M Besse B Cadranel J Pérol M Mennecier B Bigay-Game L Overall survival with crizotinib and next-generation ALK inhibitors in ALK-positive non-small-cell lung cancer (IFCT-1302 CLINALK): a French nationwide cohort retrospective study. Oncotarget (2017) 8:21903–17. 10.18632/oncotarget.15746 28423535 PMC5400633

[147] Pailler E Oulhen M Borget I Remon J Ross K Auger N Circulating tumor cells with aberrant ALK copy number predict progression-free survival during crizotinib treatment in ALK-rearranged non-small cell lung cancer patients. Cancer Res (2017) 77:2222–30. 10.1158/0008-5472.CAN-16-3072 28461563

